# TGFβ-blockade uncovers stromal plasticity in tumors by revealing the existence of a subset of interferon-licensed fibroblasts

**DOI:** 10.1038/s41467-020-19920-5

**Published:** 2020-12-09

**Authors:** Angelo L. Grauel, Beverly Nguyen, David Ruddy, Tyler Laszewski, Stephanie Schwartz, Jonathan Chang, Julie Chen, Michelle Piquet, Marc Pelletier, Zheng Yan, Nathaniel D. Kirkpatrick, Jincheng Wu, Antoine deWeck, Markus Riester, Matt Hims, Felipe Correa Geyer, Joel Wagner, Kenzie MacIsaac, James Deeds, Rohan Diwanji, Pushpa Jayaraman, Yenyen Yu, Quincey Simmons, Shaobu Weng, Alina Raza, Brian Minie, Mirek Dostalek, Pavitra Chikkegowda, Vera Ruda, Oleg Iartchouk, Naiyan Chen, Raphael Thierry, Joseph Zhou, Iulian Pruteanu-Malinici, Claire Fabre, Jeffrey A. Engelman, Glenn Dranoff, Viviana Cremasco

**Affiliations:** 1 Immuno-Oncology, Novartis Institutes for BioMedical Research, 250 Massachusetts Ave, Cambridge, MA 02139 USA; 2 Oncology, Novartis Institutes for BioMedical Research, 250 Massachusetts Ave, Cambridge, MA 02139 USA; 3 Oncology Translational Research, Novartis Institutes for BioMedical Research, 250 Massachusetts Ave, Cambridge, MA 02139 USA; 4 Biotherapeutic and Analytical Technologies, Novartis Institutes for BioMedical Research, 250 Massachusetts Ave, Cambridge, MA 02139 USA; 5 Oncology Data Science, Novartis Institutes for BioMedical Research, 250 Massachusetts Ave, Cambridge, MA 02139 USA; 6 PKS Oncology, Novartis Institutes for BioMedical Research, 250 Massachusetts Ave, Cambridge, MA 02139 USA; 7 Chemical Biology and Therapeutics, Novartis Institutes for BioMedical Research, 250 Massachusetts Ave, Cambridge, MA 02139 USA; 8 Translational Clinical Oncology, Novartis Institutes for BioMedical Research, 250 Massachusetts Ave, Cambridge, MA 02139 USA

**Keywords:** Tumour immunology, Immunotherapy

## Abstract

Despite the increasing interest in targeting stromal elements of the tumor microenvironment, we still face tremendous challenges in developing adequate therapeutics to modify the tumor stromal landscape. A major obstacle to this is our poor understanding of the phenotypic and functional heterogeneity of stromal cells in tumors. Herein, we perform an unbiased interrogation of tumor mesenchymal cells, delineating the co-existence of distinct subsets of cancer-associated fibroblasts (CAFs) in the microenvironment of murine carcinomas, each endowed with unique phenotypic features and functions. Furthermore, our study shows that neutralization of TGFβ in vivo leads to remodeling of CAF dynamics, greatly reducing the frequency and activity of the myofibroblast subset, while promoting the formation of a fibroblast population characterized by strong response to interferon and heightened immunomodulatory properties. These changes correlate with the development of productive anti-tumor immunity and greater efficacy of PD1 immunotherapy. Along with providing the scientific rationale for the evaluation of TGFβ and PD1 co-blockade in the clinical setting, this study also supports the concept of plasticity of the stromal cell landscape in tumors, laying the foundation for future investigations aimed at defining pathways and molecules to program CAF composition for cancer therapy.

## Abstract (web summary)

Understanding the tumor microenviroment is important before it can be exploited therapeutically. Here, the authors use single cell sequencing to study stromal cells in mouse tumors and identify a subset of interferon-licensed cancer associated fibroblasts that appear after anti-TGFβ treatment.

## Introduction

The tumor microenvironment is a complex ecosystem of various cellular elements that include cancer cells, immune cells, and stroma. Interactions among these cellular components, their products and the surrounding tissue are critical to create and maintain a permissive soil for tumor cells to grow^1–8^. In particular, cancer-associated fibroblasts (CAFs) have emerged as integral factors of cancer pathogenesis, owing to their abundance and to the multiplicity of processes they govern in tumors. CAFs have been implicated in many aspects of cancer development, including tumor initiation, progression and metastatic dissemination^9–15^, and increased numbers of CAFs in cancer patients are often associated with unfavorable prognosis^16,17^. One of their major functions is the synthesis of collagens, together with secretion of a palette of fibrillar proteins, ADAMs and MMPs, leading to the formation of densely reticulated networks of fibers and progressive tissue stiffening characteristic of the desmoplastic reaction in tumors. This unbalanced matrix deposition is an essential step in tumor growth and metastatic dissemination^18–21^. Furthermore, CAFs secrete a gamut of soluble factors that favor aggressive tumor cell behaviors, including epithelial to mesenchymal transition^22,23^, invasiveness^24^, and even chemoresistance^25–29^, driving cancer cell growth and cancer progression. More recently, CAFs have been implicated in immunotherapy failure, highlighting a broader role for these cells in cancer^30–37^. The extent and mechanisms underlying CAF-imposed immunomodulation are still under debate. Many of the immunomodulatory effects of CAFs are thought to involve cellular crosstalk through the secretion of inflammatory mediators. However, CAFs may also promote a tolerogenic microenvironment via shaping the density and composition of matrix components. Different studies have indeed highlighted how the spatial localization and migration properties of inflammatory cells are significantly influenced by the infrastructure created by CAFs, implicating CAFs in T cell exclusion from the tumor parenchyma and lack of response to checkpoint blockade agents^38–41^. In spite of this evidence, conflicting data suggest an opposite role for stromal elements in restraining, rather than promoting, tumor progression^42,43^. A potential explanation for this apparent paradox comes from the existence of functionally divergent populations of CAFs in the tumor microenvironment, regulating different aspects of tumor biology and response to therapy. In light of this, relative changes in fibroblast content may have contradictory effects depending on the specific composition and features of specific tumors, as well as differences in fibroblast targeting methodology. A better understanding of which specific CAF populations should be targeted, and how to achieve this task, is likely to come from a deeper appreciation of the full diversity of the tumor microenvironment. Only recently has the development of novel experimental and analytical platforms sufficiently increased accuracy to interrogate stromal cells in different organs and across different diseases, paving the way for the methodical assessment of CAFs in their physiological contexture. In this study, we employ our previously established methods for the manipulation of rare stromal cells, and combine them with cutting-edge technologies including single cell RNAseq and spatial transcriptomics to define functional connotations associated with discrete CAF phenotypes, revealing both predicted features and newly described traits. By using TGFβ-neutralizing antibodies in vivo, we also uncover the existence of a population of interferon-licensed fibroblasts with superior immunomodulatory potential. The observed changes correlate with greater responses to immune checkpoint blockade, suggesting that the CAF landscape is a plastic entity that can be harnessed for cancer therapy.

## Results

### Single cell RNAseq analysis reveals the presence of distinct subsets of cancer-associated fibroblasts

**Fig. 1:**
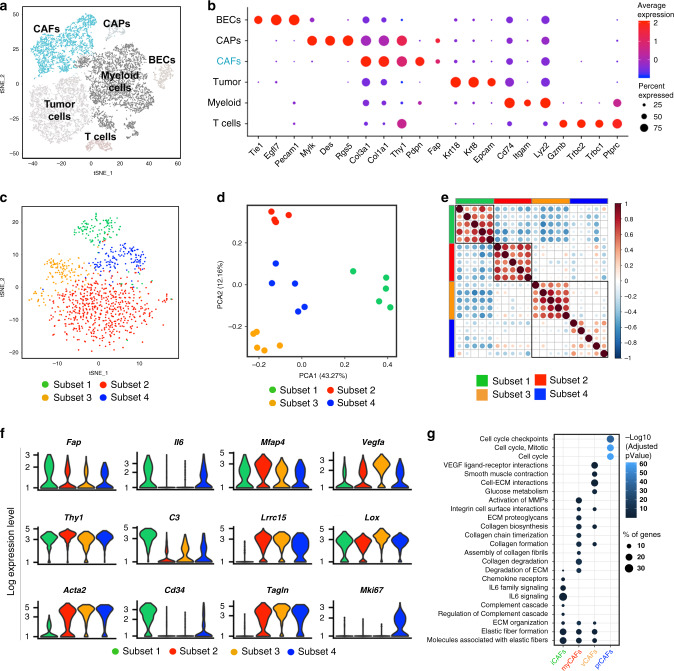
Phenotypic and functional heterogeneity of CAFs. **a** t-SNE plot of single cell RNAseq data generated from 4T1 tumors. The cluster identified as CAFs is highlighted in teal. **b** Bubble plot outlining the expression of canonical lineage cell markers utilized for cell cluster annotation. **c** CAF-only t-SNE projection showing emergent sub-clusters. **d** Biplot of Principle Components 1 and 2 from analysis of genes with highest variability (s.d. >=5), each dot represents a CAF subset from one of five independent samples. **e** Pearson correlation analysis of CAF subsets from each sample. **f** Violin plots depicting log expression level of selected genes. **g** Pathway enrichment analysis was performed on genes differentially expressed by each CAF subset using gProfiler (log(FC) > 1, Reactome pathways with *p*-value <0.05 were selected for, Fisher’s one-tailed test was used to calculate *p*-values, and *p-*values were adjusted for multiple testing). *n* = 5 samples from 3 independent experiments.

Recent work from our lab has demonstrated the existence of phenotypically and functionally divergent populations of tumor mesenchymal cells, unveiling a previously unappreciated dichotomy of the FAP^+^ stromal cell compartment^44^. More recently, observations from both preclinical and clinical samples have indicated an even higher degree of heterogeneity within tumor stroma, supporting the existence of distinct subsets of fibroblasts in tumors. The exact functional traits of different fibroblast subsets, however, remain elusive. To gain a deeper appreciation of fibroblast heterogeneity in the tumor microenvironment, single cell transcriptomic analysis was applied to stromal-enriched samples from 4T1 murine breast carcinomas, a tumor model characterized by high stromatogenic response and immune exclusion. Briefly, 6 week-old Balb/c female mice were subcutaneously inoculated with 4T1 cells; when tumors reached 700 mm^3^ in volume, they were excised and dissociated following the protocol we recently described for tumor stromal isolation^44^. Single cell suspensions were stained with antibodies against CD31 and CD90, followed by positive enrichment using Easysep beads (Supplementary Fig. 1a, b). Samples were loaded onto a 10x Genomics microfluidics chip for single cell barcoding, and sequenced using HiSeq technology. Sequencing data were aligned and processed by Cell Ranger to create UMI-annotated matrices, and libraries were filtered according to three quality control metrics (Supplementary Fig. 1c). Samples from different replicates were found to equally contribute to each of the clusters, ruling out potential batch effects (Supplementary Fig. 1d).

t-Stochastic Neighbor Embedding (t-SNE), a nonlinear, unsupervised dimensionality reduction algorithm, was used to display discrete clusters of tumor-associated cell populations (Fig. 1a) that were annotated based on the expression of canonical cell marker genes (Fig. 1b and Supplementary Fig. 1e, f). Mesenchymal cells were identified by lack of endothelial (*Pecam1*, encoding for CD31) and hematopoietic (*Ptprc*, encoding for CD45) cell markers, and by expression of mesenchymal genes such as *Thy1* and *Fap*. Expression of *Pdpn* was used to further distinguish between CAFs (*Fap*^+^*Pdpn*^+^) and cancer-associated pericytes (CAPs, *Fap*^+^*Pdpn*^-^), as previously described^44^. To further characterize the transcriptional diversity of tumor fibroblasts, barcoded events annotated as CAFs were subsequently isolated and projected by t-SNE, revealing the co-existence of four discrete sub-clusters harboring distinct gene expression profiles at basal state (Fig. 1c). Principal component analysis (PCA) based on the most highly variable genes (s.d. >= 5, measured by variance-stabilizing transformation) highlighted the significant degree of divergence between subsets, with a greater separation between Subset 1 and the other three CAF subsets (Fig. 1d). These relationships were also evident on matrix plots of population concordance, as measured by calculating the Pearson’s correlation and Euclidean distances before performing hierarchical clustering of these genes (Fig. 1e).

Analysis of the expression pattern of genes associated with specific fibroblast functions revealed unique traits of each CAF subsets (Fig. 1f). In particular, while expression of prototypical fibroblast markers such as *Fap* and *Thy1* was conserved among the four subsets, genes associated with specific fibroblast functions were found to be differentially enriched in certain clusters, indicating the existence of a functionally divergent CAF landscape. Expression of *Acta2*, the gene encoding the myofibroblast marker alpha smooth-muscle actin (αSMA), delineated one striking dichotomy between CAF subsets. Accordingly, *Acta2* was detected in three of the CAF subsets, which made up the majority of the overall CAF population, but was not found to be expressed in Subset 1, suggesting limited cell contractility potential in this subset. On the other hand, Subset 1 displayed an enrichment for genes such as *Il6* (interleukin 6), *C3* (complement 3) and *Cd34*, as well as other molecules that have been previously associated with inflammatory fibroblasts both in tumors and in other settings of pathological inflammation (Fig. 1f and Supplementary Fig. 1g)^45–47^. Among the *Acta2*^+^ CAF subsets, a high degree of diversity was discerned. Subset 2, for instance, was characterized by greater expression of genes associated with ECM remodeling, including *Mfap4*, *Lrrc15* and *Tagln*. High expression of the proangiogenic molecule *Vegfa* was detected in Subset 3, together with abundant transcripts for the collagen crosslinking protein LOX. This subset lacked, however, expression of canonical pericyte gene markers (Supplementary Fig. 1g), which were instead reported in a subset of *Vegf*-expressing stromal cells recently characterized by Bartoschek and colleagues^48^. Expression of *Mki67* was restricted to Subset 4, as well as other markers associated with cell proliferation (Supplementary Fig. 1g), suggesting that this subset may represent a proliferating population of fibroblasts in the tumor microenvironment. Distinctive subsets of CAFs were also detected in murine colorectal and kidney carcinoma, as well as melanoma (Supplementary Fig. 2), in line with recent reports from a series of studies in both animals and human tumors^37,46,48–50^, suggesting that degrees of CAF heterogeneity may exist in a broad spectrum of cancers. This heterogeneity was also appreciable at the level of protein expression. In particular, the markers *Dpp4* (CD26) and *Ly6c1* (Ly6C) were found to be highly expressed in Subset 1 both at the gene and protein level (Supplementary Fig. 3a, b), and together with αSMA staining were successfully used to differentiate CAF main subsets by flow cytometric analysis. This dichotomy was similarly observed in 4T1 tumors grown orthotopically in the mammary fat pad, as well as B16 melanomas and MC38 colorectal carcinomas (Supplementary Fig. 3c–e). Likewise, a similar phenomenon was recently described in fibroblasts from human pancreatic and breast carcinoma^37,51^, suggesting some resemblance across different murine and human tumor types.

### CAF heterogeneity reflects functional specialization

To gain more insights into the subset-specific gene programs, we performed differential gene expression analysis to define genes specifically distinguishing each CAF population. Using a log(fold change) >0.58, an adjusted *p*-value of <0.05 for significance and a minimum of 10% for expression in each cell type, we searched for genes that distinguished the four different subsets (Supplementary Fig. 4a), identifying 36 to 143 genes specifically upregulated in each class of CAFs. The differentially expressed genes were used for pathway enrichment analysis to determine functional signatures defining each subset (Fig. 1g). Consistent with the expression of inflammatory markers such as *Il6* and *Il33* (Fig. 1f and Supplementary Fig. 4a), Subset 1 was significantly enriched for pathways associated with inflammation and immune cell regulation, including cytokines and chemokine signaling as well as complement- and TNF- related pathways. Given this expression profile, it is possible that this cluster may possess major immunomodulatory potential by recruiting and modulating the activity of immune cells in the tumor microenvironment. A strong signature for pathways related to ECM deposition and interaction was depicted in Subset 2 (Fig. 1g), suggesting that these cells may have important roles in the organization of the matrix makeup that constitutes the tumor frameworks. Accordingly, the overall expression of genes encoding various collagen proteins was significantly higher in Subset 2 as compared to other subsets (Supplementary Fig. 4a–c). On the other hand, matrix remodeling molecules of the ADAM and MMP families were more uniformly expressed across CAF subsets (Supplementary Fig. 4b, c), suggesting that while enhanced collagen fiber deposition may be a defining trait of Subset 2, ECM remodeling may be a property shared by many fibroblasts. Analysis of Subset 3 and Subset 4 also highlighted important characteristics of these two subsets. Subset 3 was found to be enriched for pathways related to metabolic regulation, as marked by the glycolysis and carbon metabolism signatures (Fig. 1g). In addition, the Hif1α signature was also depicted in Subset 3, and genes associated with the overall hypoxia response were also found to be highly expressed (Supplementary Fig. 4b, c), suggesting that response to hypoxia may prompt activation of the cell energy generation machinery in this CAF population. Finally, Subset 4 was dominated by the presence of cell-cycle related signatures (Fig. 1g), and expressed many genes associated with cell-cycle progression. This finding was consistent with the unique expression of proliferative markers in Subset 4 (Fig. 1f and Supplementary Fig. 1g) and supports the notion that this population represents a subset of proliferating fibroblasts in the tumor microenvironment. Importantly, this subset clustered separately from other subsets even after cell cycle effects were regressed out from the analysis (Supplementary Fig. 4d), suggesting low transcriptional overlap with the other CAF populations and supporting the hypothesis that divergence of Subset 4 is likely not a result of cell state. Overall, these data highlight heterogeneity among CAFs, suggesting that specific fibroblast subsets are differentially poised to influence tumor growth through non-redundant functions. Based on the observed phenotypic and functional subset-specific features, the four described subsets were thereafter referred to as inflammatory CAFs (iCAFs, Subset 1), canonical myofibroblasts (myCAFs, Subset 2), VEGF^+^CAFs (vCAFs, Subset 3) and proliferating CAFs (prCAFs, Subset 4).

### TGFβ-blockade perturbs fibroblast activity in the tumor microenvironment

**Fig. 2:**
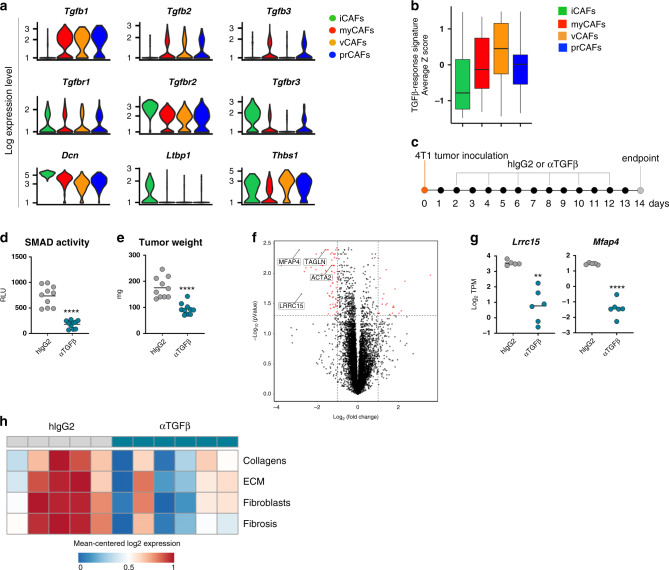
TGFβ-neutralization modulates CAF function in the TME. **a** Violin plots of genes encoding ligands, receptors, and modulators of TGFβ signaling in CAF subsets from 4T1 tumors, as analyzed by single cell RNAseq. *n* = 5 samples from 3 independent experiments. **b** Box plots depicting alignment of subset gene expression to TGFβ responsiveness signature, described previously^52^. The bounds of the boxes indicate the 25^th^ and 75^th^ percentiles, the center band reflects the median, the lower whisker indicates the minimum, and the upper indicates the maximum. **c** Study schematic of TGFβ-blockade in vivo. **d** Cell based SMAD-reporter assay confirming systemic inhibition of TGFβ activity in mice receiving TGFβ-neutralizing antibodies. Each dot represents a mouse. Mean is depicted. *n* = 10 mice. Data are representative of three independent experiments. ****p < 0.0001 (unpaired, two-tailed *t*-test with Welch’s correction). **e** Tumor weights at endpoint. Each dot represents a mouse. Mean is depicted. *n* = 10 mice per group. Data are representative of three independent experiments. ****p < 0.0001 (unpaired, two-tailed *t*-test with Welch’s correction). **f** Tumors as in **c** were pulverized and RNA was extracted for RNAseq analysis. Volcano plot of bulk RNAseq data depicting changes in gene expression between treatments; horizontal dashed line indicates an adjusted *p*-value of 0.05, vertical dashed lines indicate an absolute log2 fold change of 1 (*p*-values were calculated based on a *t*-statistic for coefficients from a linear model fit to the data). **g** Comparison of log2 TPM from bulk RNAseq data for selected genes in isotype- and anti-TGFβ-treated mice. Each dot represents a mouse. Mean is depicted. *n* = 5 (hIgG2) or 6 (αTGFβ) mice per group. Data are representative of two independent experiments. **p = 0.0013; ****p < 0.0001 (unpaired, two-tailed *t*-test with Welch’s correction). **h** Heatmap of stroma-associated signatures in bulk RNAseq data. Values were TPM normalized, log2 transformed, and row mean centered (*z*-score). Each column represents a mouse.

The coexistence of phenotypically and functionally divergent CAF populations in the tumor microenvironment raises the possibility that subset-specific traits may originate from alternative responses to microenvironmental cues. In line with this hypothesis, emerging data from pancreatic cancer have recently highlighted different growth factors that can likely contribute to the diversification of CAFs in tumors. Among these, TGFβ has been suggested to play a pivotal role in shaping the CAF landscape, especially in light of its connection to myofibroblast differentiation and function in tumors and in other fibrotic diseases^35,49,52–57^. The relationship between TGFβ and the different CAF subsets, and the implications for immune fitness and anti-tumor immunity remain to be determined. Analysis of TGFβ ligands, receptors and signaling modulators showed distinctive expression patterns among CAF subsets (Fig. 2a), reinforcing the idea that TGFβ may differently influence each CAF population. In particular, genes encoding TGFβ proteins were found to be predominantly expressed by *Acta2*^+^ subsets (myCAFs, vCAFs and prCAFs), suggesting that these CAF populations are likely to constitute the major fibroblast source of TGFβ in the TME. TGFβ receptors were found to be more generally expressed by all CAFs, although at variable levels. Notably, expression of *Dcn* (decorin) was significantly higher in iCAFs, as compared to other subsets. Given that TGFβ1 has been reported to reduce *Dcn* expression in fibroblasts, and *Dcn* itself has been shown to negatively regulate TGFβ signaling and its profibrotic effects^37,46,49^, it is likely that increased levels of *Dcn* may indicate reduced response to TGFβ in iCAFs. Accordingly, analysis of established TGFβ response signatures^52^ across each CAF subset confirmed the comparatively low level of TGFβ-induced signaling in iCAFs (Fig. 2b). iCAFs also displayed high expression of *Ltbp1* (Latent transforming growth factor beta binding protein 1) and *Thbs1* (Thrombospondin 1), suggesting a potential role in regulating availability of latent TGFβ forms.

Taken together, these data prompted us to investigate the relative effects of TGFβ-blockade on CAF phenotypes and functions in vivo. To this end, 6 week-old female mice were inoculated with 4T1 tumor cells, and subsequently treated with TGFβ-blocking antibodies, or isotype controls, following the study design depicted in Fig. 2c. Neutralization of TGFβ activity was monitored in serum using a cell-based SMAD reporter assay (Supplementary Fig. 5a). In this assay, active TGFβ induces a signaling cascade resulting in translocation of SMAD proteins to the nucleus, binding to an engineered CAGA-box, and luciferase signal. Addition of serum from mice treated in vivo with isotype control antibodies resulted in detectable luciferase signal (Fig. 2d), consistent with the presence of bioactive TGFβ. On the other hand, luciferase signal was greatly reduced when CAGA-Luc cells were stimulated with serum from mice that received anti-TGFβ treatment in vivo, supporting target engagement and neutralization of TGFβ bioactivity. Blockade of TGFβ in vivo was safe (Supplementary Fig. 5b) and sufficient to moderately delay 4T1 tumor progression (Fig. 2e and Supplementary Fig. 5c). This delay in tumor progression did not appear to stem from a direct effect of anti-TGFβ on tumor cells (Supplementary Fig. 5d, e), and was more likely a result of the microenvironmental changes following TGFβ-blockade. Indeed, administration of TGFβ-neutralizing antibodies led to appreciable differences in global gene expression, as evidenced by bulk RNAseq analysis of dry-frozen tumors (Fig. 2f). In particular, tumors from mice receiving TGFβ-blocking antibody displayed a substantial attenuation in the expression of genes associated with myofibroblast activity and extracellular matrix, including *Acta2*, *Mfap4*, and *Lrrc15* (Fig. 2f, g). In line with these findings, we depicted a substantial reduction in the overall pro-fibrotic program in tumors from anti-TGFβ-treated mice, as demonstrated by analysis of gene signatures associated with collagen deposition and fibrosis (Fig. 2h). Altogether, these data indicate that targeting TGFβ affects fibroblast dynamics in the tumor microenvironment.

### TGFβ-blockade alters tumor collagen deposition and formation of reticular fibers

**Fig. 3:**
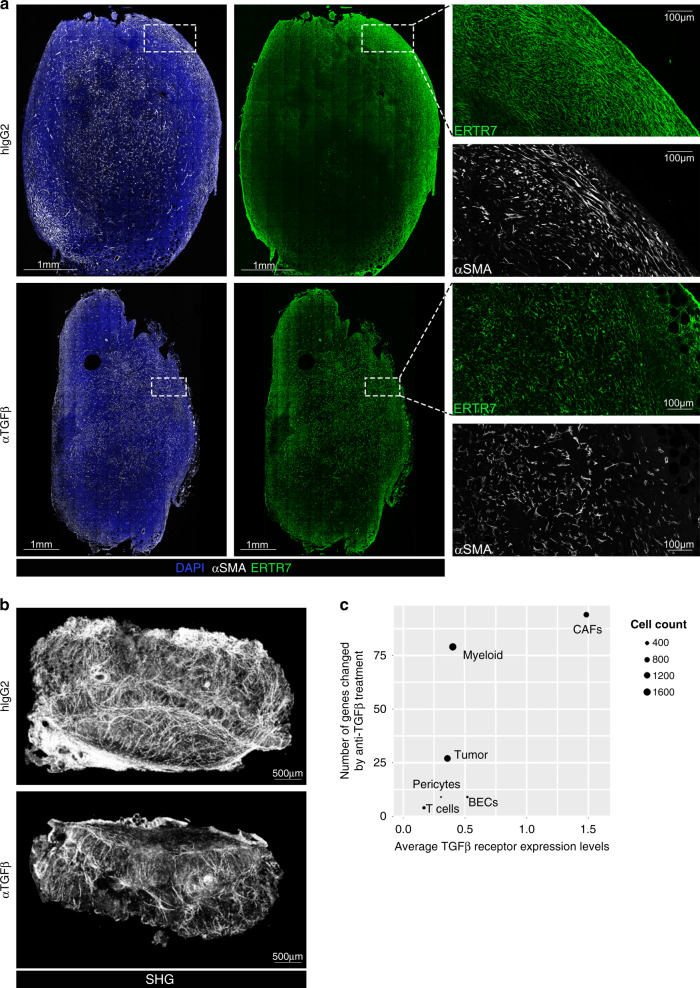
TGFβ-neutralization affects the tumor ECM architecture. **a** Cryopreserved 4T1 tumors were sectioned, stained with Dapi and antibodies against ERTR7 and αSMA, and subjected to confocal microscopic analysis. Insets show an enlargement of the boxed area. Images are representative of 6 samples from two independent experiments. **b** Collagen fibers were visualized with second-harmonic imaging microscopy. **c** Analysis of the magnitude of the transcriptional changes induced by anti-TGFβ treatment in vivo on cell populations identified in 4T1 by single cell RNAseq, plotted against the overall TGFβ receptor expression of each cell cluster. *n* = 4 mice per group from 2 independent experiments.

Given the observed changes in gene expression, we sought to determine whether TGFβ-blockade led to qualitative impairments in the formation of the structures that constitute the tumor architectural framework. To this end, cryopreserved tumors from isotype- or anti-TGFβ-treated mice were sectioned, stained, and imaged by confocal microscopy. Mirroring changes in *Acta2* expression, staining for αSMA depicted a significant alteration of the myofibroblast network after treatment with TGFβ-blocking antibodies, with a paucity of elongated, spindle-shaped cells and decreased reticular organization of the fibers comprising the extracellular matrix (Fig. 3a). Analysis of ERTR7, a marker for reticular fibers, also revealed noticeable changes in the organization of intratumoral structures, illustrating a loss of the highly reticulated network of fibers in mice treated with TGFβ-blocking antibodies (Fig. 3a).

The global impairment in the formation of the collagen framework was further documented by whole organ, serial two-photon tomography of second harmonic generation signals, as shown in Fig. 3b. Accordingly, 4T1 tumors from isotype-treated mice appeared to be characterized by the presence of dense collagen structures following an organized pattern of alignment in a dominant orientation. In contrast, the overall fibrillary collagen content was visibly reduced in tumors from mice that received TGFβ-blocking antibodies, with dispersed fibers arranged in disorganized bundles, demonstrating that TGFβ-blockade impairs myofibroblast functions, leading to destabilization of the tumor collagen framework. The overall changes in the fibroblast compartment were likely a result of a direct effect of TGFβ-blockade on these cellular elements. Indeed, single cell RNAseq analysis of tumors from isotype- or anti-TGFβ-treated mice, generated using the sample processing and data analysis pipeline described before, showed that the number of genes affected by anti-TGFβ treatment was significantly greater in CAFs as compared to other cell types (Fig. 3c), also consistent with previous reports^35,53,58^.

### TGFβ-blockade leads to loss of myCAFs and the appearance of CD73^+^ fibroblasts

**Fig. 4:**
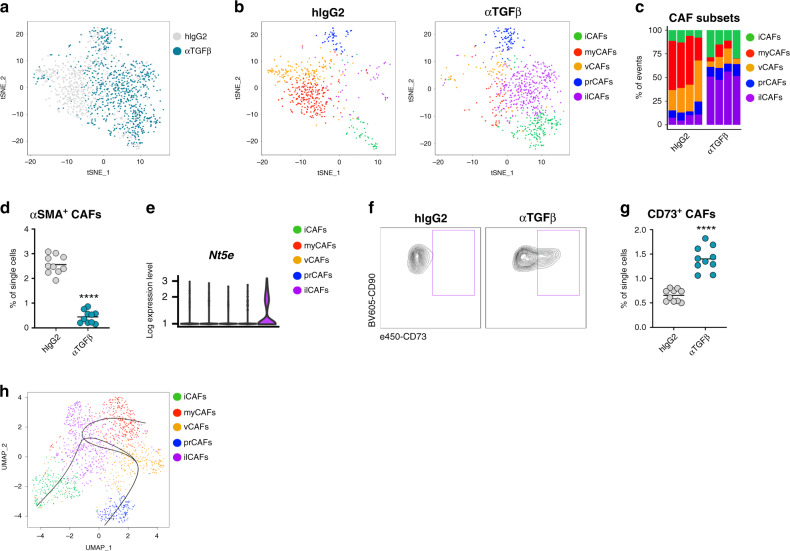
TGFβ-blockade ablates myofibroblasts and induces expansion of CD73^+^ CAFs. **a** Superimposed t-SNE projections of CAF-curated single cell RNAseq data from isotype- and anti-TGFβ-treated mice. *n* = 4 mice per group from 2 independent experiments. **b** Representative t-SNE plots highlighting CAF clusters in isotype- and anti-TGFβ-treated mice. **c** Frequency of CAF subsets within the overall CAF cluster. Each column represents a mouse. *n* = 4 mice per group from 2 independent experiments. **d** Quantification of CAFs expressing αSMA from dissociated 4T1 tumors of isotype- and anti-TGFβ-treated mice by flow cytometric analysis. Each dot represents a mouse. Mean is depicted. *n* = 10 mice per group. Data are representative of four independent experiments. ****p < 0.0001 (unpaired, two-tailed *t*-test with Welch’s correction). **e** Violin plot depicting expression of *Nt5e* in ilCAFs. *n* = 4 mice per group from 2 independent experiments. **f** Representative flow cytometric analysis of CD73 expression on CAFs from dissociated 4T1 tumors from isotype- and anti-TGFβ-treated mice. **g** Quantification of CAFs expressing CD73 as a frequency of single cells from dissociated 4T1 tumors of isotype- and anti-TGFβ-treated mice by flow cytometric analysis. Each dot represents a mouse. Mean is depicted. *n* = 10 mice per group. Data are representative of four independent experiments. ****p < 0.0001 (unpaired, two-tailed *t*-test with Welch’s correction). **h** Pseudotime analysis overlaid on UMAP projection depicting divergent trajectories among the five CAF subsets. Each dot represents a cell, and each curve represents a lineage.

Given the finding that TGFβ blockade resulted in major consequences on fibroblast activity and tumor matrix architecture, we then sought to ascertain whether TGFβ may differentially affect the discrete CAF subsets. To this purpose, we determined the relative transcriptional changes induced by TGFβ-neutralization in each of the CAF subsets. As anticipated, TGFβ-neutralization induced significant changes in the overall CAF gene expression programs, as seen by the visual shift of the overall CAF populations in the t-SNE space in mice receiving the TGFβ-neutralizing antibodies (Fig. 4a). Upon sub-clustering the CAFs, a noticeable loss of myCAFs and vCAFs was observed (Fig. 4b, c), suggesting that these subsets may be particularly sensitive to TGFβ in the tumor microenvironment. Quantification of the frequencies of αSMA-expressing CAFs using flow cytometric analysis also revealed a significant reduction in the numbers of myofibroblasts (Fig. 4d), suggesting that TGFβ- blockade may preferentially target cancer-associated myofibroblasts. Disappearance of myCAFs was not secondary to defective differentiation of myofibroblasts in the presence of TGFβ-neutralizing antibody, as treatment of established tumors in which myofibroblasts were already depicted at the time of antibody administration produced a similar loss of myCAFs (Supplementary Fig. 5f–i).

Strikingly, loss of myofibroblasts upon TGFβ-blockade was contrasted by the appearance of a transcriptionally unique population of CAFs (thereafter referred to as interferon-licensed CAFs, ilCAFs, Fig. 4b, c in purple), revealing a broader plasticity of the mesenchymal stromal compartment of the tumor microenvironment. ilCAFs were easily distinguishable from other CAF subsets by their increased gene expression of the ectonucleotidase *Nt5e* (Fig. 4e), with a similar upregulation observed for the protein encoded by *Nt5e*, CD73 (Fig. 4f). Using this marker, it was possible to track emergence of ilCAFs in tumors from mice receiving TGFβ-neutralizing antibody by flow cytometry (Fig. 4g).

It is not known, at this time, whether ilCAFs arise from reprogramming of existing myofibroblasts, or if they emerge from an alternative fate trajectory of mesenchymal progenitors in the absence of TGFβ signaling. While pseudotime analysis of transcriptional dynamics predicted divergent differentiation trajectories amongst CAF subsets (Fig. 4h), it remains possible that ilCAFs and myCAFs represent two functional states of the same existing CAF population. Further studies are required to validate this hypothesis and firmly establish the lineage relationship of each CAF cluster.

### Fibroblasts generated upon TGFβ-blockade are interferon-licensed CAFs

**Fig. 5:**
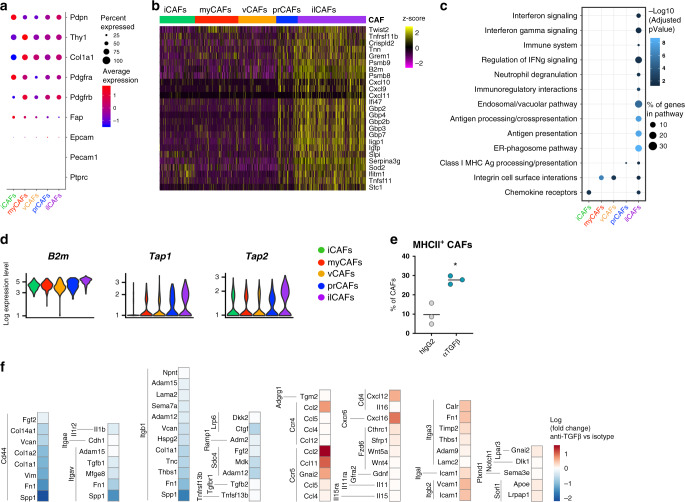
ilCAFs exhibit an immunomodulatory transcriptomic signature. **a** Bubble plot outlining the expression of the indicated cell markers in the different CAF subsets. **b** Expression heatmap of the top 25 differentially expressed genes in ilCAFs. **c** Pathway enrichment analysis was performed on genes differentially expressed by ilCAFs using gProfiler (log(FC) > 1, Reactome pathways with *p*-value <0.05 were selected for, Fisher’s one-tailed test was used to calculate *p*-values, and *p*-values were adjusted for multiple testing). **d** Violin plots depicting expression of selected genes in CAF subsets. *n* = 4 mice per group from 2 independent experiments. **e** Flow cytometric analysis of MHCII expression by CAFs in isotype- and anti-TGFβ-treated mice. Each dot represents a mouse. Mean is depicted. *n* = 3 mice per group from one experiment. *p = 0.0189 (unpaired, two-tailed *t*-test with Welch’s correction). **f** The differentially expressed receptor-ligand pairs between CAFs and T cells are shown (*p-*value <0.05, calculated using Kruskal–Wallis test).

To gain more information on the functional identity of the newly identified CAF subset, we looked into the specific transcriptional features of this class. Expression of canonical markers of tumor fibroblasts, including *Thy1*, *Fap*, and *Pdpn* was indistinguishable from other CAF subsets (Fig. 5a), and ilCAFs also lacked transcripts for endothelial, hematopoietic, and epithelial cell linages, indicating a fibroblast nature. However, analysis of the differentially expressed genes in ilCAFs underlined an enrichment for a series of molecules under the control of interferon (IFN) signaling (Fig. 5b), including many members of the guanylate-binding protein (Gbp) family. In line with these findings, pathway enrichment analysis for the genes distinguishing ilCAFs from the other CAF subsets pointed to the upregulation of IFN signaling responses in ilCAFs, with a potentiation of pathways regulated by IFN levels, including antigen processing and presentation (Fig. 5c). Genes encoding for MHC proteins and MHC-related molecules were also found to be highly expressed in ilCAFs (Fig. 5b–d), and an upregulation of MHCII protein was detected in CAFs after TGFβ-blockade in vivo (Fig. 5e). The enrichment for IFN-responsive genes encoding chemoattractants for antigen-experienced T cells, including CXCR3 ligands *Cxcl9*, *Cxcl10* and *Cxcl11*, was also observed (Fig. 5b), indicating that ilCAFs may have essential roles in directing T cell trafficking within tumors. Consistent with this hypothesis, analysis of the ligand-receptor interactions depicting CAF-T cell communication identified several gene pairs involved in T cell recruitment and migration that were significantly upregulated by TGFβ neutralization (Fig. 5f).

To ascertain whether differences in regional progenitors and secretomes may affect the stromal microenvironment, we analyzed the response to TGFβ-neutralization in orthotopic 4T1 tumors. To this end, 4T1 tumor cells were inoculated in the mammary fat pad of Balb/c female mice and animals were treated with TGFβ-neutralizing antibodies or isotype controls, using the same dose and regimen as previously employed for s.c. tumors (Supplementary Fig. 6a). Similar to the 4T1 s.c. setting, orthotopic 4T1 tumor progression was modestly delayed by TGFβ-blockade (Supplementary Fig. 6b). Orthotopic tumors were then collected and processed, and single cell RNAseq was performed using the same workflow and QC parameters as for s.c. tumors. As shown in supplementary Fig. 6c, the same cell populations described in 4T1 s.c. tumors were also found in orthotopic tumors, with the addition of a cluster of mammary cells characterized by expression of genes such as *Krt18* and *Slpi*. Projection of CAF-only events from orthotopic tumors further supported the comparability between the two sites, revealing the existence, at steady state, of fibroblast subsets with overlapping features to those described for CAFs from s.c. tumors (Supplementary Fig. 6d, e). Moreover, TGFβ-blockade produced the characteristic loss of myCAFs/vCAFs, as observed in s.c. tumors, while expanding other CAF subsets, with a subset showing clear features indicative of response to IFN, including an upregulation of interferon-inducible genes such as CXCR3 ligands, IRFs and Gbp molecules (Supplementary Fig. 6d–f). The additional expansion of another CAF subset lacking myofibroblastic features in anti-TGFβ- treated orthotopic tumors might stem from differences in the regional availability of interferon in the mammary fat pad, and may indicate a loss of specialized functionality in the absence of interferon-induced transcriptional activity. This is also consistent with the expression of *Nt5e* at the gene level in other CAF subsets in orthotopic tumors (Supplementary Fig. 6f). Nonetheless, the striking analogy with the data from the s.c. model was evident at the protein level, with a clear loss of αSMA staining and a concomitant upregulation of CD73 in CAFs from orthotopic tumors treated with anti-TGFβ (Supplementary Fig. 6g–i). These findings support the hypothesis that heterogeneity of CAFs and their response to TGFβ may represent generalized phenomena that are independent from the source of fibroblasts themselves (likely from surrounding subdermal tissue in s.c. tumors, and mammary tissue for orthotopic tumors).

To further validate the effects of TGFβ-blockade on human CAF dynamics, we tested TGFβ-neutralizing antibodies on freshly dissociated samples of human microsatellite-stable colorectal carcinomas (MSS-CRC), a desmoplastic tumor type that is poorly responsive to immunotherapy. Ex vivo cultures established from gently dissociated tumor tissues were analyzed by single cell RNAseq. Among CAFs, a high degree of heterogeneity could also be ascertained, with some correlation with the subset phenotypes described in murine tumors (Supplementary Fig. 7a, b). Samples were then treated with recombinant human TGFβ1, in the presence or absence of TGFβ-blocking antibodies, and changes in gene expression were depicted. In these conditions, TGFβ-blockade induced a measurable transcriptional modulation in different cell types, with pronounced changes observed in CAFs (Supplementary Fig. 7c). Analysis of the most differentially regulated genes in CAFs revealed some parallelism to the observations generated in 4T1 tumors. In particular, a downregulation of genes associated with fibroblast activity and ECM was already detected after 24 hours of treatment, pointing to a reduction of myofibroblast features in human CAFs. Even more importantly, despite the fact that discrete genes were found to be differentially upregulated in each sample, these genes were overall indicative of an IFN-response signature induced in CAFs in all the samples analyzed, underscoring biological similarities that go beyond the observed and expected inter-patient variability (Supplementary Fig. 7d). Notably, *Nt5e* was found to be expressed in some human CAF subsets (Supplementary Fig. 7b) but was not among the most differentially upregulated genes after 24 hours of anti-TGFβ treatment (Supplementary Fig. 7d), suggesting that genes associated with the IFN-response machinery are induced rapidly after TGFβ-blockade in CAFs, while phenotypic changes may require more time.

Altogether, these data demonstrate that TGFβ-blockade leads to appreciable remodeling of the tumor fibroblast landscape in mice and humans, exposing the induction of a unique CAF population marked by distinct responses to IFN and considerable immunomodulatory potential.

### TGFβ-blockade augments T cell infiltration and activation

**Fig. 6:**
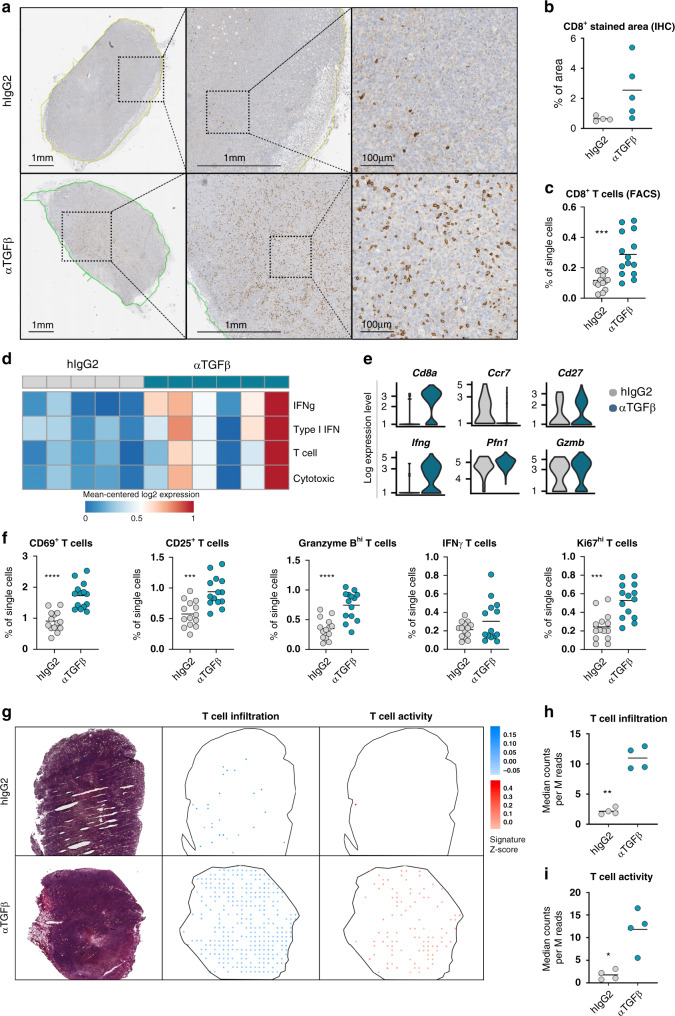
TGFβ-blockade promotes T cell infiltration and activation. **a** Paraffin-embedded sections of tumors from isotype- and anti-TGFβ-treated mice were stained for CD8 and analyzed using immunohistochemistry. **b** Changes in CD8^+^ T cell infiltration were determined by quantification of CD8-stained area from IHC samples. Each dot represents a sample. Mean is depicted. *n* = 4 (hIgG2) or 5 (αTGFβ) mice from one experiment. *p* = 0.0894 (unpaired, two-tailed *t*-test with Welch’s correction). **c** Frequency of CD8^+ ^T cells was determined by flow cytometric analysis on dissociated tumors. Each dot represents a mouse. Mean is depicted. *n* = 14 mice per group. Data are representative of three independent experiments. ***p = 0.0006 (unpaired *t*-test with Welch’s correction). **d** Heatmap of T cell-associated signatures as expressed in bulk RNAseq data from isotype- and anti-TGFβ-treated mice. Values were TPM normalized, log2 transformed, and row mean centered (z-score). Each column represents a mouse. **e** Violin plots depicting expression of selected genes in T cells from single cell RNAseq data. *n* = 4 mice per group from 2 independent experiments. **f** Frequency of T cell expression of activation markers was determined by flow cytometric analysis on dissociated tumors. Each dot represents a mouse. Mean is depicted. *n* = 14 mice per group from one experiment. *p*-values for each marker are as follows: CD69 ****p < 0.0001; CD25 ***p = 0.0002; Granzyme B ****p < 0.0001; IFNγ *p* = 0.1756; Ki67; ***p = 0.001 (unpaired, two-tailed *t*-test with Welch’s correction). **g** Tumor tissues were sectioned and subjected to spatial transcriptomic analysis. Dots are indicative of enrichment for T cell presence (blue) or activity (red) representative in that particular area. One representative of 4 mice is depicted for each treatment group. **h**, **i** The signature score for the indicated parameters was calculated across the tissue section and graphed. Each dot represents a mouse. Mean is depicted. *n* = 4 mice per group from two independent experiments. **p = 0.0014; *p = 0.0192 (unpaired *t-*test with Welch’s correction).

The remodeling of the CAF landscape after TGFβ neutralization, and in particular the appearance of a CAF subset with potential T cell modulatory activity, prompted us to determine the effects of TGFβ-blockade on the establishment of anti-tumor immune responses. In line with the heightened gene expression of T cell chemotactic molecules in ilCAFs, immunohistochemical staining for CD8 depicted a considerable difference in the infiltration of cytotoxic T cells between treatment groups (Fig. 6a). Specifically, a paucity of T cells was observed in isotype-treated tumors, in contrast to an extensive infiltration of CD8^+^ T cells in mice receiving TGFβ-neutralizing antibodies (Fig. 6a, b). A similar increase in the number of cytotoxic T cells upon TGFβ-blockade was corroborated using flow cytometric analysis of dissociated tumors (Fig. 6c). The greater number of T cells upon TGFβ-blockade was not merely a result of changes in tumor size, as a significant increase of both CD8^+^ T cells and in general of CD3^+^ lymphocytes was still ascertained when numbers where normalized by mass of tumors or single cells (Supplementary Fig. 8a, b). Similarly, no correlation between T cell numbers and tumor weights was detected (Supplementary Fig. 8c). Transcriptomic analysis of gene signatures associated with T cell effector functions also depicted an augmentation of T cell functional activity in tumor samples from anti-TGFβ-treated mice (Fig. 6d), supporting the idea that TGFβ-blockade may result in overall awakening of immune cell responses. These findings were substantiated by examination of the expression of canonical T cell markers at the single cell level. Specifically, increased *Cd8* expression in T cells from tumors of mice that received TGFβ-blocking antibodies was accompanied by higher levels of the activation marker *Cd27* in conjunction with a downregulation of *Ccr7* (Fig. 6e), thereby indicating an effector phenotype. Expression of *Ifng*, and to a lesser extent *Pfn1* and *Gzmb*, was also upregulated in T cells following TGFβ-blockade (Fig. 6e), illustrating their enhanced activity on a per-cell basis. These findings were corroborated by flow cytometry, as a significant increase in the frequency of T cells bearing an effector phenotype was observed (Fig. 6f and Supplementary Fig. 8d–f). In order to assess the extent of this heightened activation in respect to the tissue distribution of the infiltrating T cells, we then generated a tumor roadmap for signature genes collectively associated with the presence of T lymphocytes and their activity by employing spatial transcriptomic analysis. In line with the findings illustrated before, isotype control-treated mice showed a paucity of tumor infiltrating T cells (Fig. 6g, h), with negligible activity (Fig. 6g, i). On the other hand, greater T cell infiltration in tumors of mice receiving TGFβ-neutralizing antibodies was accompanied by visible and homogeneous activity throughout the entire parenchyma (Fig. 6g–i), suggesting the reversal of spatial and functional immune exclusion in these tumors.

### TGFβ neutralization augments the efficacy of PD1 immunotherapy in vivo

**Fig. 7:**
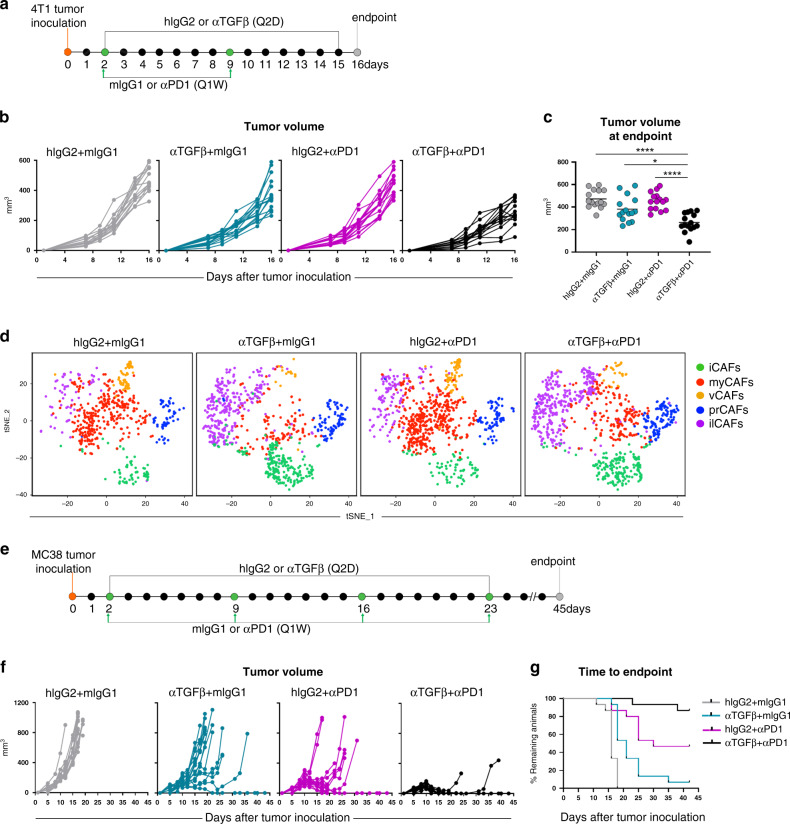
TGFβ-blockade augments the efficacy of PD1 immunotherapy. **a** Study schematic of TGFβ/PD1 co-blockade in 4T1 tumor bearing mice. **b** Individual 4T1 tumor volumes for animals treated as in **a** were determined by measurement with calipers. Each line represents a mouse. *n* = 15 mice per group. Data are representative of four independent experiments. **c** 4T1 tumor volumes measured at endpoint, each dot represents a mouse. Mean is depicted. *n* = 15 mice per group. Data are representative of four independent experiments. *p*-values were calculated using Brown-Forsythe and Welch ANOVA with Dunnett’s T3 multiple comparisons test, and individual values are as follows: hIgG2 + mIgG1 vs αTGFβ + mIgG1, *p* = 0.0883; hIgG2 + mIgG1 vs hIgG2 + αPD1, *p* = 0.9768; hIgG2 + mIgG1 vs αTGFβ + αPD1, ****p < 0.0001; αTGFβ + mIgG1 vs αTGFβ + αPD1, *p = 0.0155; hIgG2 + αPD1 vs αTGFβ + αPD1, ****p < 0.0001. **d** t-SNE plots of CAF subsets separated by treatment group. *n* = 4 mice per group from two independent experiments. **e** Study schematic of TGFβ/PD1 co-blockade in MC38 tumor bearing mice. **f** Individual MC38 tumor volumes for animals treated as in **e** were determined by measurement with calipers. Each line represents a mouse. **g** Mice treated as in **e** were removed from study when tumors reached 900 mm^3^, and the fraction of animals remaining on study was depicted using a Kaplan-Meier curve. One representative of 4 experiments is shown in **f** and **g**, *n* = 15 mice per group per experiment.

The data described so far demonstrate that TGFβ blockade shapes the fibroblast landscape in tumors, unleashing IFN signaling in CAFs and boosting the infiltration and activation of cytotoxic T cells. As these changes are predicted to contribute to the generation of productive anti-tumor immunological responses, we tested whether TGFβ neutralization may act in synergy with checkpoint blockade immunotherapy. To this end, 4T1 tumor-bearing mice were assigned to 4 different groups, and were treated with isotype control antibodies, anti-TGFβ, anti-PD1, or a combination of the two (Fig. 7a). Body weight changes from baseline and tumor volumes were recorded at least twice a week and are illustrated in Supplementary Fig. 9a and Fig. 7b. As previously documented, anti-PD1 alone did not affect 4T1 tumor growth. However, tumor progression was notably delayed in mice receiving the combined treatment (Fig. 7b). Measurement of tumor volumes at endpoint confirmed the lack of efficacy in mice treated with anti-PD1 alone, while some single agent activity for TGFβ-blockade was noted (Fig. 7c). A greater reduction in tumor volumes was observed in mice receiving both antibodies, demonstrating that TGFβ neutralization can boost PD1 immunotherapy in this otherwise refractory tumor model (Fig. 7c).

Treatment with anti-PD1 alone did not produce appreciable changes in the overall fibroblast transcriptional program. On the other hand, remodeling of the CAF landscape and appearance of ilCAFs was evident following the combined treatment with anti-TGFβ and anti-PD1 (Fig. 7d), suggesting that the revived sensitivity to PD1-blockade in 4T1 tumors may stem, at least in part, from stromal adaptation induced by TGFβ-neutralization. Nonetheless, these changes were insufficient to drive complete regression in 4T1 tumors, in line with the notion that the generation of durable anti-tumor immune responses may require modulation of multiple steps of the cancer immunity cycle.

Based on this consideration, the combinatorial activity of TGFβ- and PD1-neutralizing antibodies was also assessed in mice inoculated with MC38, an immune-inflamed tumor type with heterogeneous responsiveness to immunotherapies. Following the study design depicted in Fig. 7e, mice were treated in vivo with isotype control antibodies, anti-TGFβ, anti-PD1, or a combination of the two, and were monitored for body weight changes (Supplementary Fig. 9b) and tumor growth (Fig. 7f). In line with other studies described before^35,53^, while anti-TGFβ and anti-PD1 alone showed modest single agent activity in MC38 (Fig. 7f), the combination of the two antibodies resulted in significant therapeutic efficacy, yielding a complete response rate of 86.6% (Fig. 7g). Importantly, TGFβ-blockade in this model led to appreciable changes in the tumor microenvironment which were indicative of stromal-immune remodeling (Supplementary Fig. 9c–e). These alterations were comparable to the microenvironmental remodeling observed in 4T1 tumors. In order to test the induction of immunological memory, mice that responded to therapy with eradicated primary tumors were re-challenged with MC38 tumor cells on the opposite flank (Supplementary Fig. 9f) and monitored for tumor re-occurrence. Under these conditions, both groups receiving anti-PD1 alone or in combination with TGFβ-blockade during the first tumor challenge exhibited appreciable tumor rejection rates (Supplementary Fig. 9g–h), suggesting that both therapeutic regimens can lead to the development of immunological memory.

Taken together, these studies implicate TGFβ-blockade and the remodeling of CAF dynamics as advantageous strategies to augment the cancer immunity cycle, supporting future studies aimed at elucidating innovative opportunities to strategically reprogram CAFs in the tumor microenvironment.

## Discussion

In the last few decades, the discovery that the immune system could be harnessed to fight tumor cells has led to the development of novel treatments for cancer. Immune checkpoint blockade, in particular, has shown clinical benefit for patients with some forms of solid tumor and hematologic malignancies^59–67^. However, there still remains a large proportion of patients that derive little to no benefit from therapies such as PD1/PDL1 blockade, highlighting the need to decipher the mechanisms that underlie immunotherapy resistance^68^. A growing body of work from preclinical models as well as data from clinical studies both point to a prominent role for cancer-associated fibroblasts in limiting the efficacy of checkpoint inhibition^35,37,53,69,70^. With the implementation of technologies for the interrogation of rare stromal cell populations, we have now started to appreciate the existence of divergent functional traits within CAFs and, more broadly, stromal cells in tumors. Building on these recent findings, our investigation improves the current taxonomy of CAFs, describing four phenotypically and functionally divergent subsets of fibroblasts in the tumor microenvironment. TGFβ-blockade also uncovers a population of CAFs that fosters an immune-permissive microenvironment, revealing a striking plasticity in the tumor stromal compartment and providing principles for therapeutic intervention.

It has been postulated that CAFs may represent a collection of cellular subsets, rather than a homogeneous entity. Different cancers, and even similar tumor types in different patients, have been shown to preferentially drive the differentiation of CAFs with specific features^46,71–74^, providing the basis for the identification of tumor-specific subtypes in certain indications^75,76^. In addition, the concept of intra-tumoral CAF heterogeneity has also started to emerge, providing an additional layer to the growing awareness of fibroblast diversity. The differential expression within the same tumor of canonical fibroblast cell markers such as FAP, podoplanin and αSMA clearly supports the simultaneous existence of phenotypically unique subpopulations^46,77^. Studies in pancreatic, breast and lung carcinomas have shown the co-existence of CAFs with contrasting CD10 expression and different roles in supporting tumor cell growth, suggesting that phenotypic differences may also indicate functional differentiation^29,73^. Similarly, the presence of a specific CAF subset has been recently shown to sustain chemoresistance in breast cancer^78^, and heterogeneity in the immunoregulatory functions of CAFs has also been described^79,80^. The holistic interrogation of tumor-associated elements with technologies such as single cell sequencing further enhanced the cellular resolution of CAFs and the tumor microenvironment^46,48,49,72,74,81–88^. Studies in non-tumor sites have also started to highlight the global heterogeneity of these cells in various organs and across different diseases, indicating that the existence of functionally diverse fibroblast subsets is not restricted to tumor tissues^89–95^.

An emerging theme from these and other studies is the identification of a dedicated population of fibroblasts with intrinsic fibrogenic properties and with superior connective tissue deposition potential in settings of fibrosis and wound repair. Similarly in tumors, we found that ECM-related functions are predominantly executed by myCAFs, the largest population of *Acta2*^+^ CAFs found in these tumors. myCAFs were found to also express genes that are upregulated in fibroblasts during fibrosis in non-neoplastic tissues, including *Postn* (Periostin) and *Tagln* (Transgelin), raising the hypothesis that the pathogenesis of intratumoral fibrosis may share some commonality with that of other fibrotic diseases^89^. Among the *Acta2*^+^ fibroblasts, a small number of cells with proliferative potential was also ascertained, similar to what recently shown by other studies in additional murine models^37,48^. Diverging from *Acta2*^+^ CAFs, the fibroblast cluster denominated as iCAFs showed some similarity to a subset of CAFs recently identified in murine and human PDAC^37,46^, including high expression of inflammatory mediators and loss of myofibroblastic exemplar genes. Consistent with the enrichment for immune-related molecules, iCAFs also showed unique expression of *Cd34*, a marker associated with immunofibroblasts and the induction of tertiary lymphoid structures in other pathological inflammatory conditions^45,94^. It has been suggested that iCAFs differentiate in tumors under the influence of IL1 signaling^49^, and we also found them to express receptors for IL1. Conversely, however, in our dataset iCAFs lacked some of the markers previously identified in IL1-driven iCAFs from PDAC tumors, including *Lif* (Leukemia inhibitory factor) and *Il11*^49^, indicating a certain level of diversity. The most remarkable finding from our study, however, was the characterization of a CAF subset arising from TGFβ-blockade, marked by prominent immunomodulatory functions. The emerging CAF subset was found to express a gamut of molecules associated with interferon signaling, and was therefore referred to as IFN-licensed CAFs (ilCAFs). One major feature associated with IFN response in ilCAFs was the expression of MHC molecules and other factors associated with the antigen processing and presentation machinery. Whether these features correlate with a functional role for ilCAFs in immune priming remains to be determined. Indeed, some literature has suggested the possibility that certain CAFs may induce T cell receptor ligation in CD4^+^ T cells^50^, but more recent studies reported these cells to be of mesothelial rather than mesenchymal origin^37,93^.

The expression of chemokines, and in particular CXCR3 ligands, is also consistent with an IFN signature in ilCAFs, and suggests that ilCAFs may be involved in T cell recruitment through the production of directional cues. Indeed, concurrent increases in T cell infiltration to the TME was observed in animals treated with anti-TGFβ. As such, we speculate that the appearance of ilCAFs upon TGFβ-neutralization may be a contributing factor in potentiating anti-tumor immunity and responsiveness to checkpoint inhibition therapy through facilitating either enhanced infiltration or activation of cytotoxic T cells. By contrast, TGFβ-neutralization resulted in substantial reduction to ECM density and decreases to myCAFs – factors which have instead been linked to immune exclusion and immunotherapy failure^35,37,53^. We thus propose that this combined shift in CAF composition ultimately alters the dynamics of tumor-infiltrating lymphocyte spatiality and activation, and facilitates the generation of functional immunity secondary to TGFβ-inhibition.

Mechanistically, the role of TGFβ signaling in shaping CAF function has been firmly established^54,55^. However, it is notable that TGFβ-neutralization appears to target only select CAF subsets, despite ubiquitous expression of TGFβ receptors. The presence of iCAFs, for instance, is largely unaffected by the loss of TGFβ signaling. This is consistent with the finding that iCAFs and myCAFs appear to follow separate differentiation trajectories, in line with what was described by Dominguez and colleagues. In particular, iCAFs showed a low degree of TGFβ responsiveness, despite expressing comparable levels of TGFβ receptors. This is notable, as previous studies have identified an essential reliance on autocrine TGFβ signaling loops in maintaining myofibroblast features^56^. A lack of TGFβ expression, in combination with a minimal TGFβ response signature, suggests that iCAFs are not actively engaged in this autocrine signaling loop, and are therefore less affected by TGFβ. In steady state conditions, the myCAFs differentiation fork dominates under the influence of TGFβ signaling, resulting in an abundance of myofibroblasts in the tumor microenvironment. TGFβ-blockade potentiates an alternative differentiation trajectory, not only restricting myofibroblast differentiation, but also promoting the development of ilCAFs, likely by reprogramming of a stromal progenitor.

We cannot exclude that the effects of TGFβ-neutralization span beyond the modulation of CAF phenotypes and functions. Given that TGFβ is known to restrict T cell proliferation through the reduction of IL-2 production, as well as downregulate expression of certain cytotoxic effector mediators^96^, it is in fact conceivable that TGFβ-neutralization may act both on stromal cells to promote T cell infiltration, and directly on tumor-infiltrating lymphocytes to enhance their activity^97^. Nevertheless, our data demonstrate that limiting TGFβ bioavailability elicits the formation of an immune-permissive microenvironment, in line with recent reports illustrating that TGFβ targeting through different methodologies allows immunological control of tumor growth in combination with checkpoint blockade therapy^35,53,98^. These findings have provided the rationale for the investigation of a TGFβ blocking antibody in combination with anti-PD1 (ClinicalTrials.gov Identifier: NCT02947165) in patients with advanced malignancies. Clinical data from the ongoing phase I/Ib trial are not available yet, but are likely to complement previous and current efforts exploring applicability of TGFβ-blockade through different modalities.

Despite the recent success of immunotherapies, we still face tremendous challenges in understanding the lack of clinical benefits in certain patients. Clues from the microenvironment, and in particular a better understanding of the stromal elements that restrain anti-tumor immunity, are likely to guide future efforts aimed at unleashing the full potential of immunotherapies.

## Methods

### Mice

Six-week-old, sex-matched Balb/c and C57Bl/6 mice were purchased from Charles River Laboratories and allowed to acclimate for 3 days prior to manipulation. Animals had access to food and water ad libitum for the entire duration of the study. Animals were monitored throughout the studies for well-being and behavior, including grooming, hunching, and ambulation. Sample sizes for in vivo experiments were determined based on previous studies with stromal cells in 4T1 tumors^44^. All animal experiments were approved by, and performed in accordance with the guidelines from the Institutional Care and Use Committee (IACUC) at Novartis Institutes for BioMedical Research (Protocol 20 IMO 035).

### Cell lines

4T1, B16 and Renca cells were obtained from American Type Culture Collection (ATCC), expanded, aliquoted, and banked in liquid nitrogen. MC38 cells were received from NCI under MTA# 38699-15, expanded, frozen and collected in the NIBR cell line repository. No additional authentication was performed on these cells. Cell lines were maintained in either RPMI 1640 (Gibco, 11875-085) or Dulbecco’s Modified Eagle Medium (Gibco, 11965-092) as appropriate, each containing 10% heat-inactivated FBS (VWR, 1500-500), 1% Penicillin-Streptomycin (Gibco, 15140-122), and 1% l-glutamine (Gibco, 25030-81) for one week prior to implant. Prior to inoculation into recipient animals, cell lines were tested and found to be free of mycoplasma and viral contamination in the IMPACT VIII PCR assay panel (IDEXX BioResearch, Missouri).

### Tumor models

On the day of tumor inoculation, cells were detached using 0.25% trypsin, resuspended in sterile PBS, and implanted subcutaneously on the animal upper-right dorsal flank at a concentration between 1 × 10^5^ and 1 × 10^6^ cells in 100 μL (4T1 and Renca into Balb/c mice; MC38 and B16 into C57Bl/6). For orthotopic tumors, 1 × 10^5^ 4T1 cells in 50 μL of PBS were implanted into the third mammary fat pad. Mice were monitored for tumor growth and changes in body weight and body condition at least 2 times/week. Mice were euthanized when one of the following humane endpoints was reached: body weight loss equal or greater than 20% from baseline; tumor volume exceeding 800 mm^3^; presence of ulcerated tumors; poor body condition score.

### In vivo therapeutic treatment

TGFβ-neutralizing antibodies were described before^99^. For in vivo experiments, mice were dosed intraperitoneally with TGFβ-neutralizing antibody or human IgG2 isotype control at a final concentration of 10 mg/kg (Q2D starting either 2 or 7 days after tumor inoculation as indicated). For combination studies, PD-1 antibody or mouse IgG1 isotype control were given intravenously at a final concentration of 10 mg/kg (Q1W starting 2 days after inoculation).

### Tumor digestion

Tumors were collected and digested as previously described^44^. Briefly, tissues were minced into fine pieces (approximately 1 mm^3^), transferred into 15 mL conical tubes containing 2 mL of digestion buffer [RPMI (Gibco), 2% FBS, 0.2 mg/mL Collagenase P (Roche), 0.2 mg/mL Dispase (Gibco), and 0.1 mg/mL DNase I (Roche)], and placed into a water bath at 37 °C. Tissue fragments were subjected to consecutive cycles of agitation/pipetting, and the supernatant containing freed cells was collected every 20 minutes and quenched at 4 °C in 50 mL conical tubes containing cold flow cytometry buffer (PBS, 2% FBS, and 2 mmol/L-EDTA). When the digestion was completed, cells were filtered through a 70-μm mesh, centrifuged and used for subsequent analyses.

### Flow cytometric analysis

Tumor single-cell suspensions were resuspended in flow cytometry buffer (PBS, 2% FBS, and 2 mmol/L EDTA), blocked with Fc block, and then stained with different fluorochrome-conjugated antibodies for 15 min on ice. The following antibodies were used at the noted diluitions: anti-CD31 (Biolegend 102506, 1:200), anti-αSMA (Sigma C6198, 1:100), anti-CD8 (eBioscience 25-0081-82, 1:100), anti-FoxP3 (eBioscience 61-5773-82, 1:50), anti-CD11b (Biolegend 101228, 1:100), anti-PDPN (Biolegend 127410, 1:100), anti-CD4 (BD 563790, 1:100), anti-CD45 (BD 564279, 1:600), anti-CD45.2 (BD 564880, 1:400), anti-CD90 (Biolegend 140317, 1:100), anti-CD3 (Biolegend 100229, 1:100), anti-CD26 (eBioscience 45-0261-82, 1:100), anti-Ly6c (Biolegend 128026, 1:100), anti-I-A/I-E (Biolegend 107632, 1:100), and anti-CD73 (Biolegend 127217, 1:100). Samples were run on a BD flow cytometry Fortessa instrument and analyzed with FlowJo.

### Stromal enrichment

Single cells from the tumor digestion were resuspended in mouse FC block (Miltenyi #130-092-575). Biotinylated CD31 (Biolegend 390, 1:50) and Thy1 (Biolegend 53-2.1, 1:50) antibodies were used for positive selection, using the EasySep Selection Kit (Stemcell Technologies #18559). Cells were then washed in cold PBS, counted, and resuspended to approximately 10^6^ for sequencing.

### Single cell RNAseq

The 10x Genomics Chromium Single Cell 3’ Reagent v2 kit (cat. no. PN-120-236) was used with standard conditions and volumes to process cell suspensions for 3’ transcriptional profiling. The cell suspension volumes were calculated for a target cell recovery of 6000 cells, and loaded on the Chromium per manufacturer’s guidelines. The resultant purified cDNAs were quantified on the Agilent Tapestation using the High Sensitivity D1000 ScreenTapes (cat. no. 5067-5584) and Reagents (cat. no. 5067-5585). The final single cell 3’ libraries were quantified using the Agilent High Sensitivity D5000 ScreenTapes (cat. no. 5067-5592) and Reagents (cat. no. 5067-5593). The libraries were diluted to 10 nanomolar in Qiagen Elution Buffer (Qiagen material number 1014609), denatured, and loaded on the Illumina MiSeq® at 12 picomolar with the MiSeq Reagent Kit v3 (cat. no. MS-102-3001) to assess sample quality and loading normalization for the HiSeq4000. The normalized libraries were loaded at a range of 2.5 to 4.0 picomolar on an Illumina cBOT using the HiSeq® 4000 PE Cluster Kit (catalog number PE-410-1001). The single cell 3’ libraries were sequenced on a HiSeq® 4000 for 26 base pairs on the first read, followed by an 8 base pair index read, and a 98 base pair second read, using 2 HiSeq® 4000 SBS kits, 50 cycles (cat. no. FC-410-1001). All sequence intensity files were generated on instrument using the Illumina Real Time Analysis software. The resulting intensity files were demultiplexed and then aligned to the transcriptome using the 10x Genomics Cellranger software package.

### Data pre-processing

The Seurat R package was used for data preprocessing and analysis. Raw counts from the 10× Cell Ranger software were converted into Seurat objects, removing cells with <500 genes, and filtered for genes expressed by at least 0.1% of all cells. The percentage of mitochondrial genes was calculated for each cell, and those that fell above the 95^th^ percentile were accounted for as dead cells and filtered out. The gene expression measurements of each cell were then normalized by the total expression before being log-transformed. From the log-normalized data, the top 2000 most highly variable genes were identified to be included in PCA and clustering analysis. Scaling was performed by centering the expression for each gene before dividing the mean-centered expressions by the standard deviation.

### Cluster identification and annotation

Principal component analysis (PCA) was performed on the scaled data to reduce the dimensions, with number of components chosen based on a cumulative proportion (accumulated amount of explained variance) of 95%. Clusters of cells were identified by shared nearest neighbor (SNN) algorithm. Doublets were filtered out on a per-cluster basis, removing events that contained a number of genes above the 95^th^ percentile. Cell type annotations were assigned to clusters based on the expression of canonical features in a minimum percentage of cells.

### Differential gene expression

The likelihood-ratio test was used for the identification of differentially expressed genes between cell types. Genes detected in at least 10% of clusters, had an absolute fold-change >1.5, and a bonferroni adjusted *p*-value <0.05 were selected for.

### Pseudotime analysis

The Slingshot R package was used to perform pseudotime analysis on the data. Lineages were identified from previously calculated PCA embedding, and prCAFs were chosen to act as the starting cluster, or progenitor, based on findings by Dominguez and colleagues^37^. Smooth representations were constructed for each lineage, and resulting principal curves were overlaid on a UMAP of the data.

### Pathway analysis

Pathway enrichment analysis was performed on upregulated differentially expressed genes (log(fold-change) >1, adj *p*-value <0.05), which were ordered by decreasing importance, using the gProfiler program. Reactome pathways with FDR corrected *p*-values of <0.05 and significant enrichment scores were selected for further analysis.

### Spatial transcriptomic tissue imaging, library preparation, and sequencing

Libraries were prepared using the Spatial Transcriptomic Library Preparation Glass Slides following manufacturer’s instructions (manual version 180611). In brief, fresh frozen tissues embedded in OCT compound (Tissue-Tek) were sectioned at a thickness of 16 µm and placed on a Spatial Transcriptomic Library Preparation Glass Slide. Sections were fixed with formaldehyde solution 36.5–38%, stained with Hematoxylin and Eosin Y, and imaged with an Aperio Scanscope AT Slide Scanner. Sections were pre-treated with Collagenase (Thermofisher) for 20 min at 37 °C and permeabilized with 0.1% Pepsin (Sigma Aldrich) for 10 min at 37 °C. cDNA synthesis was carried out overnight using SuperScript III Reverse Transcriptase in the presence of Actinomycin. Tissue was removed by Proteinase K (Qiagen) treatment for 2 h, and probe cleavage carried out by USER (NEB) enzymatic cleavage. Array spots were imaged on a GenePix 4100 A scanner after hybridizing Cyanine-3 labeled complementary probes. Second strand synthesis and in vitro transcription was carried out on the cleaved probes using the MEGAscript T7 Transcriptions Kit (Invitrogen). The resulting amplified RNA product was then ligated to an aRNA Ligation Adapter by T4 RNA Ligase 2, truncated (NEB). The ligation product was converted to cDNA by SuperScript III Reverse Transcriptase in presence of cDNA specific primer. A final PCR reaction, using amplification and sample indexing primers, was carried out for a varying number of cycles (5−15 cycles) dependent on QPCR quantified cDNA input amounts. The resulting libraries were sequenced on an Illumina HiSeq 2500 instrument with 2 Rapid V2 SBS chemistry with paired-end reads (Read 1 26 cycles, Read 2 120 bp).

### Spatial transcriptomics analysis

Reads were mapped against the human genome (GRCh38) and Ensembl (release 85) transcripts were quantified using the ST pipeline 1.6.2^100^. Samples were next normalized using a custom analysis pipeline (http://github.com/lima1/sttkit) based on Seurat 3.1 and SCTransform^101^. Spots with fewer than 400 detected genes were excluded. Batch effects across different biological and technical replicates were removed using the Seurat 3.1 FindIntegrationAnchors and IntegrateData workflow. The number of detected genes per spot was regressed out in the normalization.

### Immunohistochemistry (IHC)

IHC was performed on formalin-fixed, paraffin embedded tissue slides on the Ventana Discovery XT Autostainer (Roche Diagnostics Corporation, Indianapolis, IN), using a monoclonal rat antibody raised against mouse CD8α (clone #4SM15, eBioscience/Thermo Fisher Scientific, Waltham, MA, 1:100). Whole slide images were captured at 20x using a Leica Biosciences Aperio AT2 slide scanner (Leica Biosystems, Buffalo Grove, IL). Graphed analysis shown was conducted using HALO AreaQuantification algorithm (Indica Labs, Albuquerque, NM) to assess CD8 staining areas.

### Confocal immunofluorescence microscopy

Tumors were excised, fixed for 2 to 4 hours in 4% paraformaldehyde, and treated in 30% sucrose overnight. Saturated samples were embedded in optimal cutting temperature medium (Sakura Tissue-Tek #4583), and cryopreserved before sectioning. 12- to 20-μm thick sections were immunostained, and imaged using a Leica SPX8 laser-scanning confocal microscope with a 40x objective. For staining, the following antibodies were used: anti-PDPN (Biolegend 127410, 1:100), anti-ERTR7 (Abcam ab51824, 1:100), anti-αSMA (Sigma C6198, 1:100), DAPI (Molecular Probes D3571), goat anti-hamster (Life Technologies A21451, 1:500), donkey anti-rat (Life Technologies A21208, 1:500).

### Second harmonic generation signal imaging

Tumors were harvested, drop-fixed in 4% paraformaldehyde in PBS for 24 h, then transferred to 4 °C 0.1% Sodium Azide in PBS. The tumors were then embedded in agarose and prepared for imaging by TissueVision on the TissueCyte 1600FC (TissueVision, Inc, Newton MA. www.tissuevision.com) using Serial Two-Photon Tomography. High resolution collagen fiber data were collected to construct second harmonic generation (SHG) images arising from endogenous contrast of collagen fibers (920 nm excitation, 20 × 1.0 NA objective, emission filter bandpass 442−478 nm). In order to image collagen deep within the sample, the microscope is equipped with a microtome on the stage so that the sample can be sectioned following collection of each 3D image stack. The imaging resolution and sectioning parameters were as follows: imaging volume = 0.93 um/pixel *x*,*y*; 2 μm *z*; 25 optical sections for total imaging depth of 50 um and physical sections = 100 μm thick; 25 sections for a total tissue depth of 2.5 mm.

### Calculation of ligand-receptor (LR) interaction scores

Potential LR interaction between cell types were quantified similar to that described earlier (MP Kumar et al, Cell Reports, 2018). Briefly, a specific LR interaction between cell type 1 and cell type 2 is computed as the product of averaged ligand expression over all cells of cell type 1 and averaged receptor expression over all cells of cell type 2. Each interaction is named under the convention: ligand, receptor, putative ligand-releasing cell type 1, putative receptor-expressing cell type 2. Interactions between control and αTGFβ conditions were identified as significantly different by Kruskal–Wallis test by rejecting the null hypothesis that the population median of the 2 groups are equal. Log fold changes (LogFC) is computed as log2 ratio of interactional values in the αTGFβ condition over control condition where 1 was added to each interaction to avoid zero denominator error.

### In vitro proliferation assay

4T1 cells were maintained in RPMI 1640 Medium (Gibco, 11875-085) containing 10% Fetal Bovine Serum (VWR, 1500-500). On the day of the assay, cells were detached using TrypLE™ Express Enzyme 1x (Gibco, 12605-010), counted and resuspended at a final concentration of 2 × 10^5^ cells/mL in RPMI 1640 with or without 10% FBS. 100uL (2 × 10^4^ cells) were plated onto an E-Plate (ACEA, 300600910) and incubated for 30 min prior to baseline measurements. The plate was then removed from the incubator and 3 ng of recombinant mouse TGFβ1 was added to some wells, together with various concentrations of TGFβ-neutralizing antibody. The plate was placed onto a XCELLigence RTCA MP machine (ACEA Biosciences, 00380601040) and read at intervals of 15 min for the first 8 h, then at 1 h intervals for the next 48 h to calculate impedance (cell index).

### In vitro culture of human tissue and assessment of TGFβ-blockade

Dissociated human colorectal carcinoma samples were purchased from Discovery Life Sciences and cultured in RPMI 1640 (Gibco, 11875-085) with 10% FBS (VWR, 1500-500). Recombinant human TGFβ1 (Peprotech, AF10021C10UG, 3 ng/mL) and TGFβ-blocking antibodies (10 μg/mL) were added to some of the wells, and cells were incubated at 37 °C. 24 hours later, plates were collected, cells recovered and processed for single cell RNAseq analysis as described above.

### Statistical analysis

Statistics used in each figure are indicated in the figure legends.

## Supplementary information

Supplementary Information

## Data Availability

The RNAseq data have been deposited in the NCBI Gene Expression Omnibus database under accession code GSE160687. The remaining data are available within the Article, Supplementary Information or available from the authors upon request. Source data are provided with this paper.

## Source data

Source Data file
