# Supplementary material for: TGFβ-blockade uncovers stromal plasticity in tumors by revealing the existence of a subset of interferon-licensed fibroblasts
Source: Nat Commun. 2020 Dec 9;11:6315. doi: 10.1038/s41467-020-19920-5 (PMC7725805; doi:10.1038/s41467-020-19920-5)
Supplement: Supplementary file 1 — Supplementary Information [file 41467_2020_19920_MOESM1_ESM.pdf]

Supplementary Information

TGFβ-blockade uncovers stromal plasticity in tumors by revealing the existence of a subset of interferon-licensed fibroblasts

Grauel et al.

Grauel et al., Supplementary Figure 1

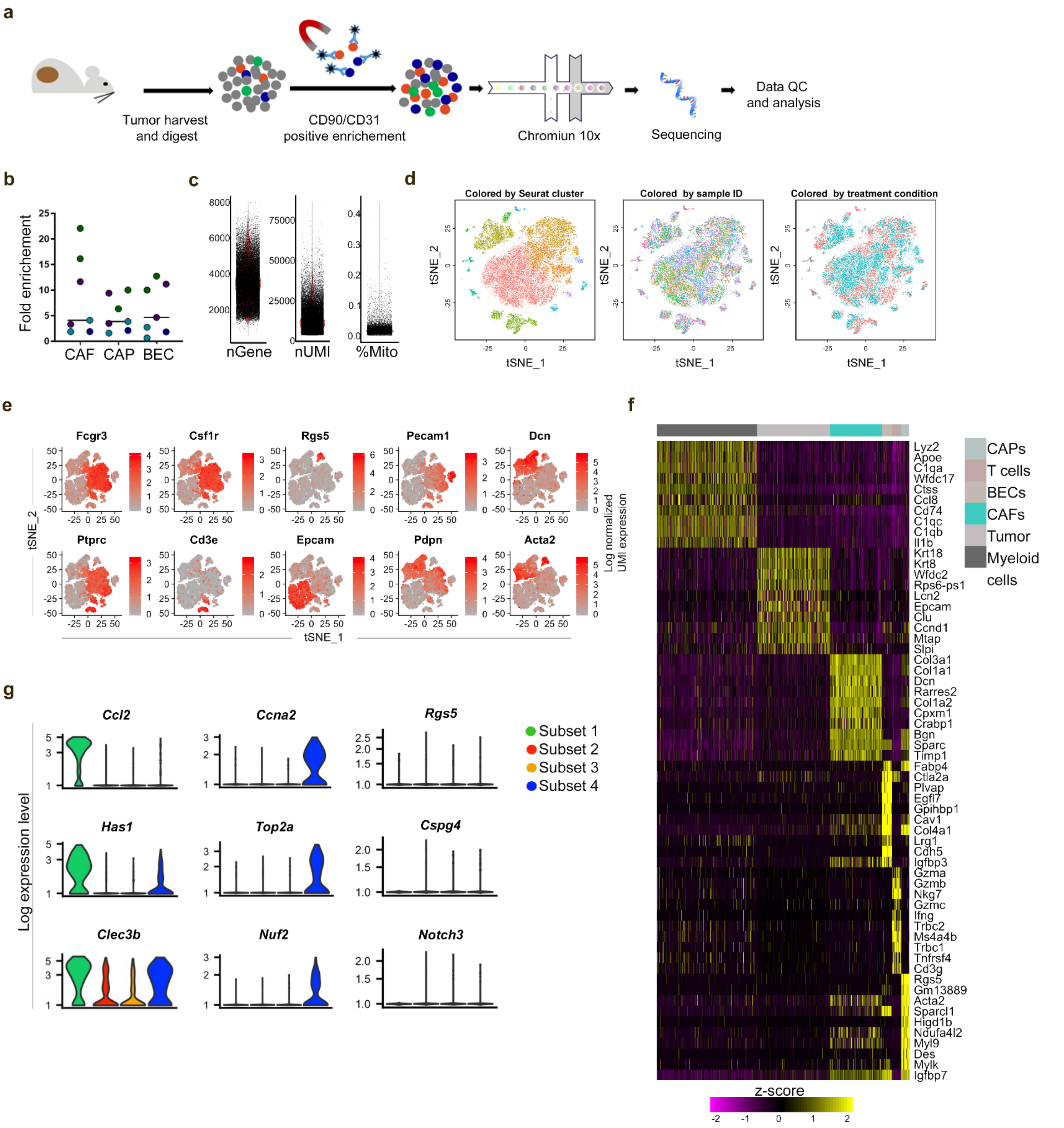

Supplementary Figure 1. Single cell RNA sequencing workflow. a. Schematic outlining the workflow for single cell RNAseq. Tumors were excised, digested, and stromal fraction was enriched using CD90/CD31 positive enrichment prior to loading on 10x chip for single cell RNAseq processing. **b.** Fold enrichment for stromal cells across different tumor types, as determined by flow cytometric analysis. Teal, 4T1; Green, CT26; Purple, MC38, Red, Renca. n=7 mice from 5 independent experiments. **c.** Violin plots showing the number of genes, UMI count, and mitochondrial content of each cell. **d.** Distribution of events based on cluster, sample of origin, and treatment. **e.** Feature plots highlighting expression of lineage cell marker genes utilized for cluster identification. **f.** Heatmap of the top 10 differentially expressed genes in each cluster. **g.** Violin plots depicting expression for the indicated genes. n=5 mice from 3 independent experiments.

Grauel et al., Supplementary Figure 2

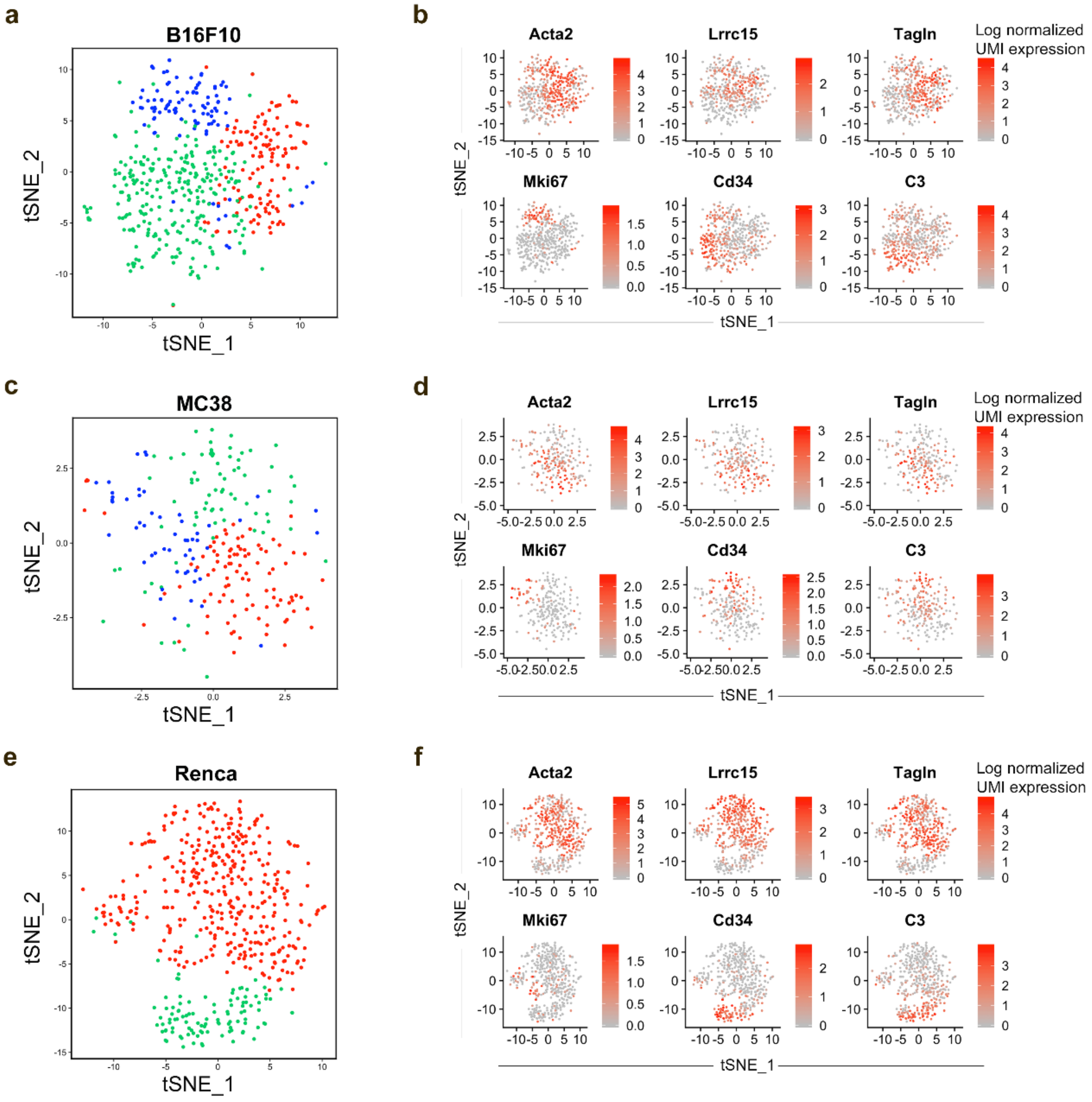

Supplementary Figure 2. CAF heterogeneity is observed in tumors of different origin. a, c, e. t-SNE plots of CAF-curated datasets from single cell RNAseq analysis of melanoma (B16F10, **a**), colorectal (MC38, **c**), and Renal cell (Renca, **e**) carcinomas. **b, d, f.** Feature-plots of selected genes in CAF-curated single cell RNAseq data from these models. n=2 mice per tumor type.

Grauel et al., Supplementary Figure 3

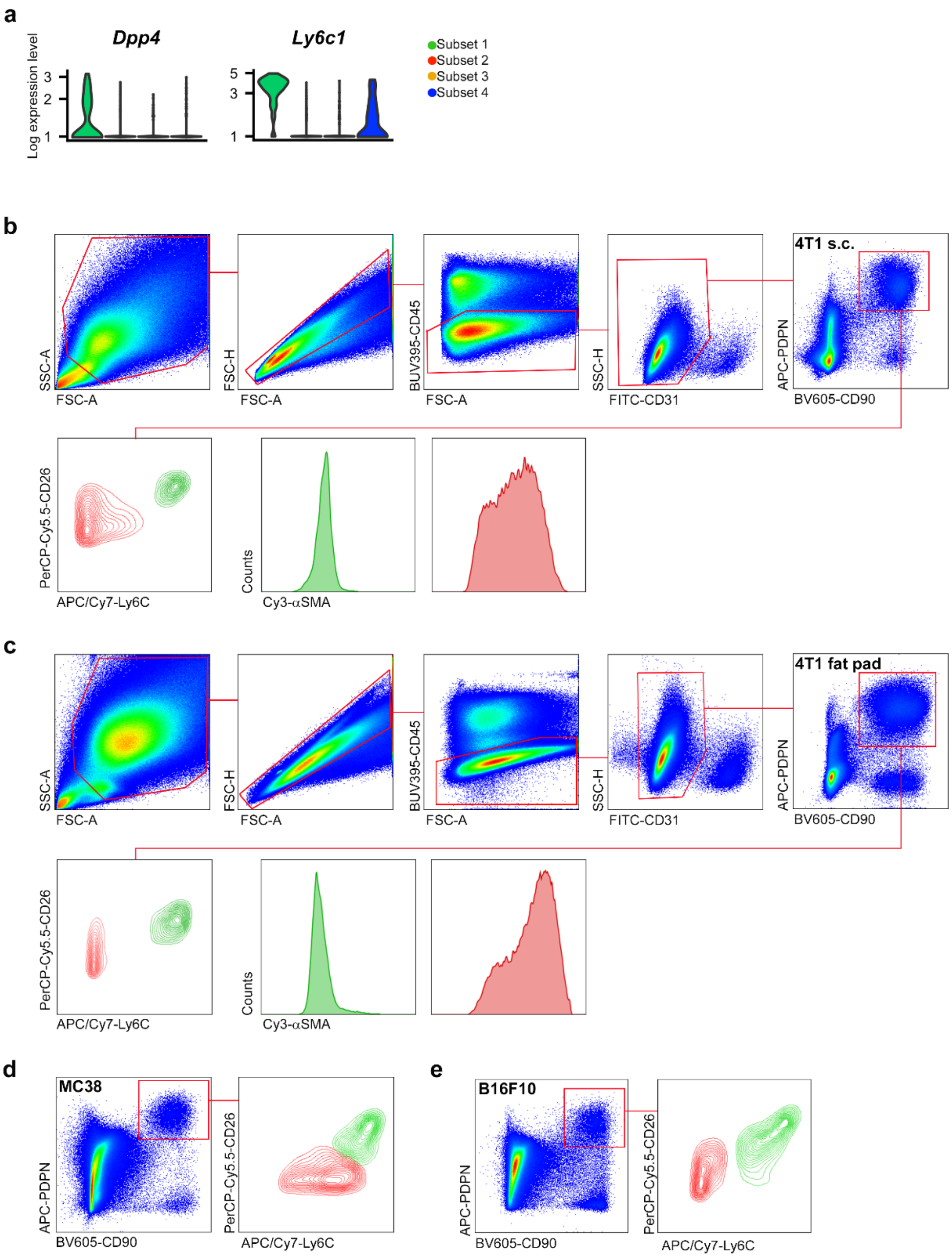

Supplementary Figure 3. CAF subset characterization. a. Violin plots of *Dpp4* and *Ly6c1* gene expression from single-cell RNAseq data in CAF subsets. n=5 mice from 3 independent experiments. **b.** Gating strategy for CAFs in subcutaneously implanted 4T1 tumors with subsetting based on CD26 (*Dpp4*), Ly6C and αSMA protein expression. **c.** Gating strategy for CAFs in orthotopically implanted 4T1 tumors with subsetting based on CD26 (*Dpp4*), Ly6C and αSMA protein expression. **d,e.** CD26 and Ly6C expression in CAFs from MC38 **(d)** and B16F10 tumors **(e)**.

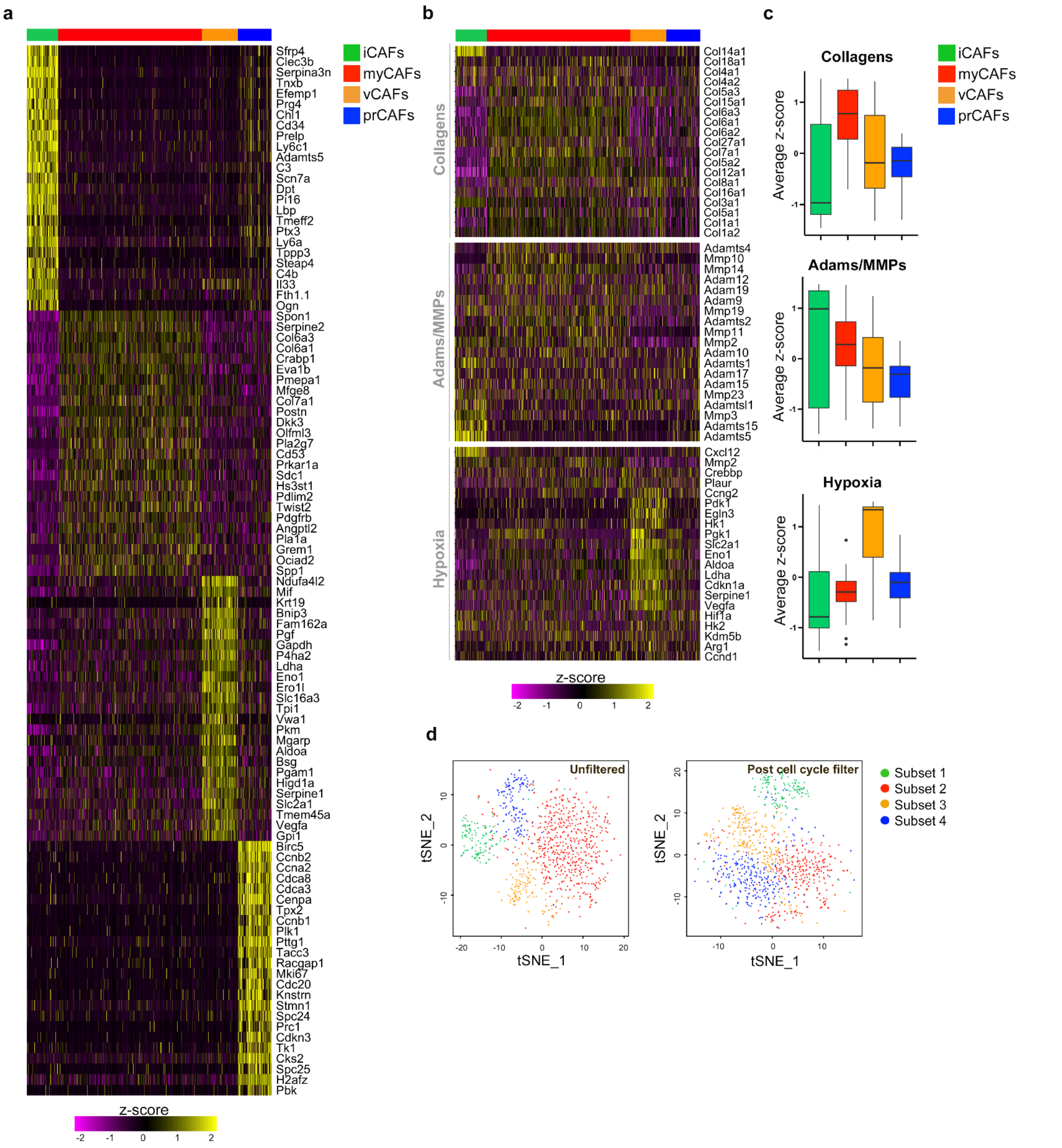

Supplementary Figure 4. CAF subsets are characterized by different functional traits. a. Heatmap highlighting the top 25 differentially expressed genes in each CAF subset. **b, c.** Heatmaps (**b**) and graphs of the z-scores (**c**) for collagen, Adam/MMP, and hypoxia-associated genes in CAF subpopulations. The bounds of the boxes indicate the 25th and 75th percentiles, the center band reflects the median, the lower whisker indicates the minimum, and the upper indicates the maximum. **d.** CAF subsets in murine 4T1 unfiltered or after regressing out cell cycle effects, colored by cluster identity. n=5 mice from 3 independent experiments.

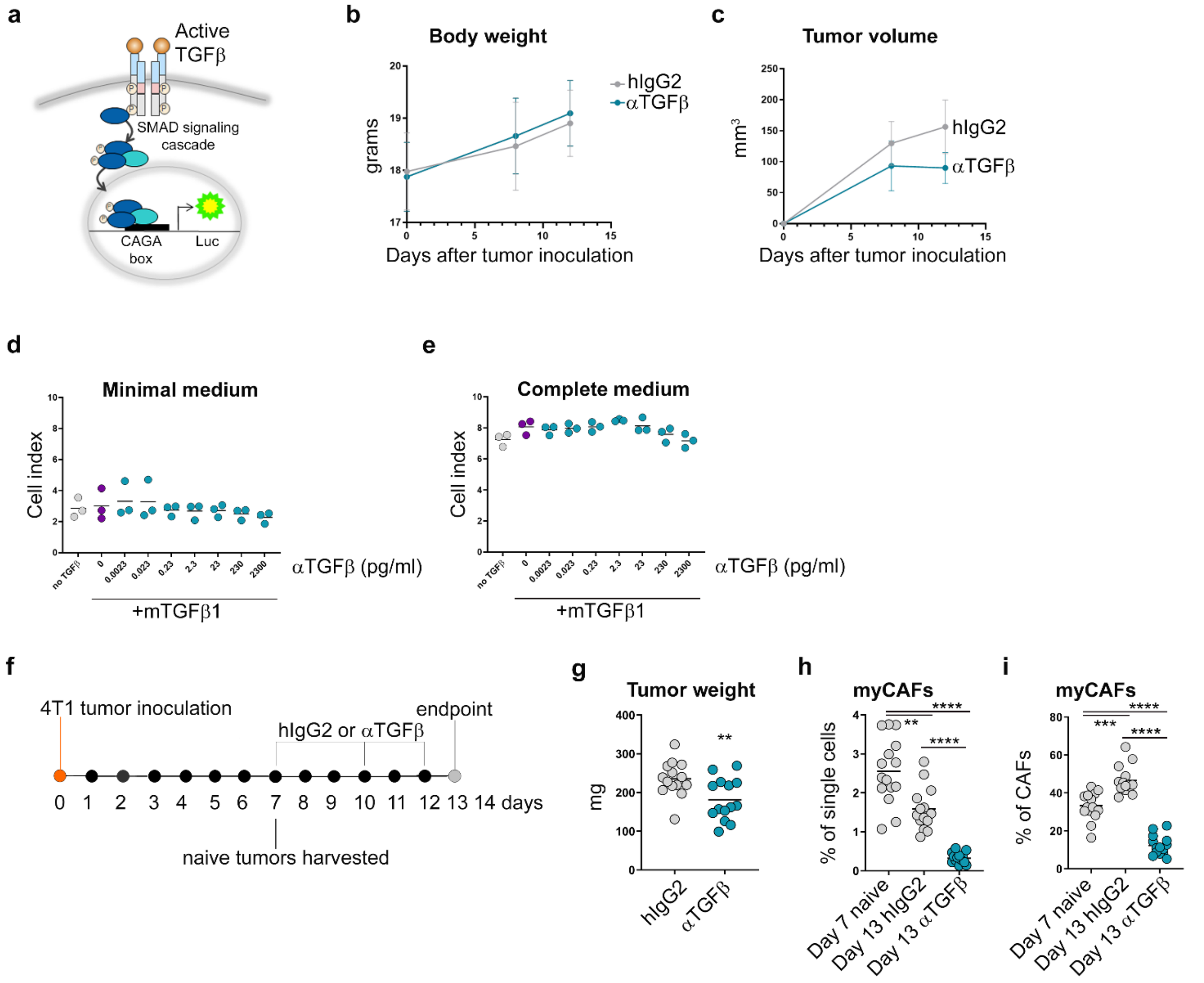

Supplementary Figure 5. TGFβ blockade is well tolerated in mice and moderately delays 4T1 tumor progression. a. Schematic of cell-based SMAD-reporter assay used to determine the presence of active TGFβ in mouse serum. **b, c.** Body weights (**b**) and tumor volumes (**c**) from 4T1 tumor-bearing mice treated with isotype or TGFβ-neutralizing antibodies were recorded throughout the study. The lines represent the mean +/- SD for each treatment group (n=10 mice per group). One representative of 3 experiments is shown. **d, e.** Cell proliferation index of 4T1 tumor cells in vitro cultured in serum-free (**d**) or complete (**e**) culture media containing recombinant murine TGFβ in a range of anti-TGFβ antibody concentrations. Each dot represents a technical replicate from one experiment. **f.** Schematic outlining study conducted with delayed dosing regimen. **g.** Tumor weights from mice treated as in (**f**) taken at end of study. Each dot represents a mouse. Mean is depicted. n=14 mice per group from one experiment. **p=0.0085 (unpaired, two-tailed t test with Welch's correction). **h, i.** Flow cytometric analysis of αSMA+ CAF frequencies from dissociated tumors taken at both timepoints as described in (**f**). Each dot represents a mouse. Mean is depicted. n=14 mice per group from one experiment. p values were calculated using Brown-Forsythe and Welch ANOVA with Dunnett's T3 multiple comparisons test, and individual values are as follows: (**h**) D13 hIgG2 vs D13 αTGFβ, ****p<0.0001; D13 hIgG2 vs D7 naive, **p=0.0035; D13 αTGFβ vs D7 naive, ****p<0.0001; (**i**) D13 hIgG2 vs D13 αTGFβ, ****p<0.0001; D13 hIgG2 vs D7 naive, ***p=0.0001; D13 αTGFβ vs D7 naive, ****p<0.0001.

Grauel et al., Supplementary Figure 6

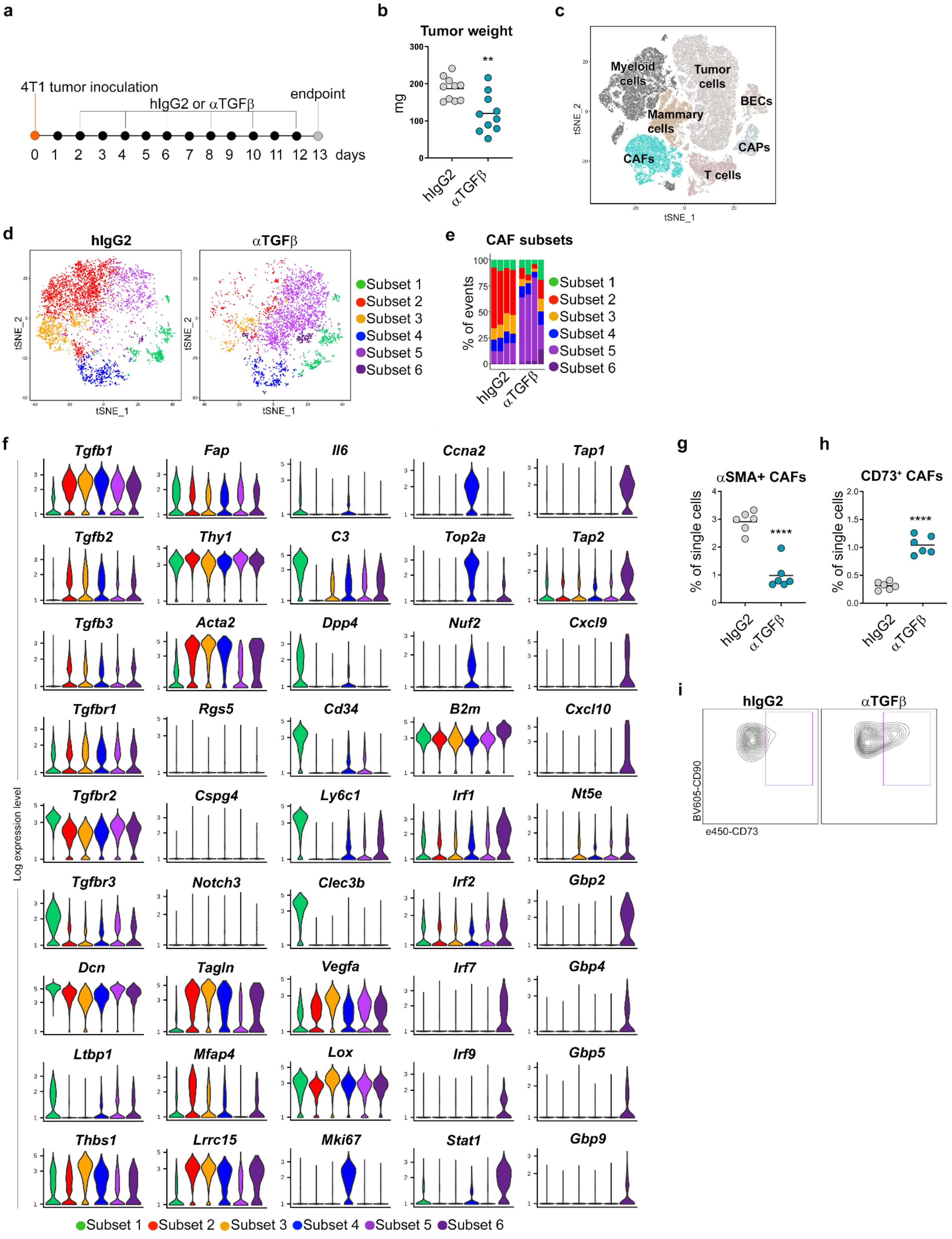

Supplementary Figure 6. Fibroblast response to TGFβ-blockade in orthotopic 4T1 tumors. a. Study schematic of TGFβ-blockade in orthotopically implanted 4T1 tumors. **b.** Tumor weights from mice treated as in (**a**) taken at end of study. Each dot represents a mouse. **p=0.0034 (unpaired, two-tailed t test with Welch's correction). **c.** t-SNE plot of single cell RNAseq data generated from 4T1 tumors. Clusters were annotated using a marker expression strategy as in Figure 1b. Mammary cells were identified through expression of genes such as *Krt18*, *Slpi*, and *Sparc*, and lacked expression of canonical hematopoietic, endothelial and mesenchymal cell markers. The cluster identified as CAFs is highlighted in turquoise. n=4 mice per group from one experiment. **d.** Representative t-SNE plots of CAF-curated dataset highlighting CAF subset clusters in isotype- and anti-TGFβ-treated mice. The colors used for different CAF clusters are adapted from those previously used in the representation of 4T1 s.c. tumors, with two shades of purple used for the CAF subsets that expanded upon anti-TGFβ treatment. n=4 mice per group from one experiment. **e.** Frequency of CAF subsets within the overall CAF cluster. Each column represents a mouse. n=4 mice per group from one experiment. **f.** Violin plots depicting log expression level of selected genes among CAF subsets identified as in (**d**). **g, h.** Quantification of CAFs expressing αSMA (**g**) and CD73 (**h**) from dissociated orthotopic 4T1 tumors of isotype- and anti-TGFβ-treated mice, assessed by flow cytometric analysis. Each dot represents a mouse. Mean is depicted. n=6 mice per group from one experiment. ****p<0.0001 (unpaired, two-tailed t test with Welch's correction). **i.** Representative flow cytometric analysis of CD73 expression on CAFs from dissociated orthotopic 4T1 tumors from isotype- and anti-TGFβ-treated mice.

Grauel et al., Supplementary Figure 7

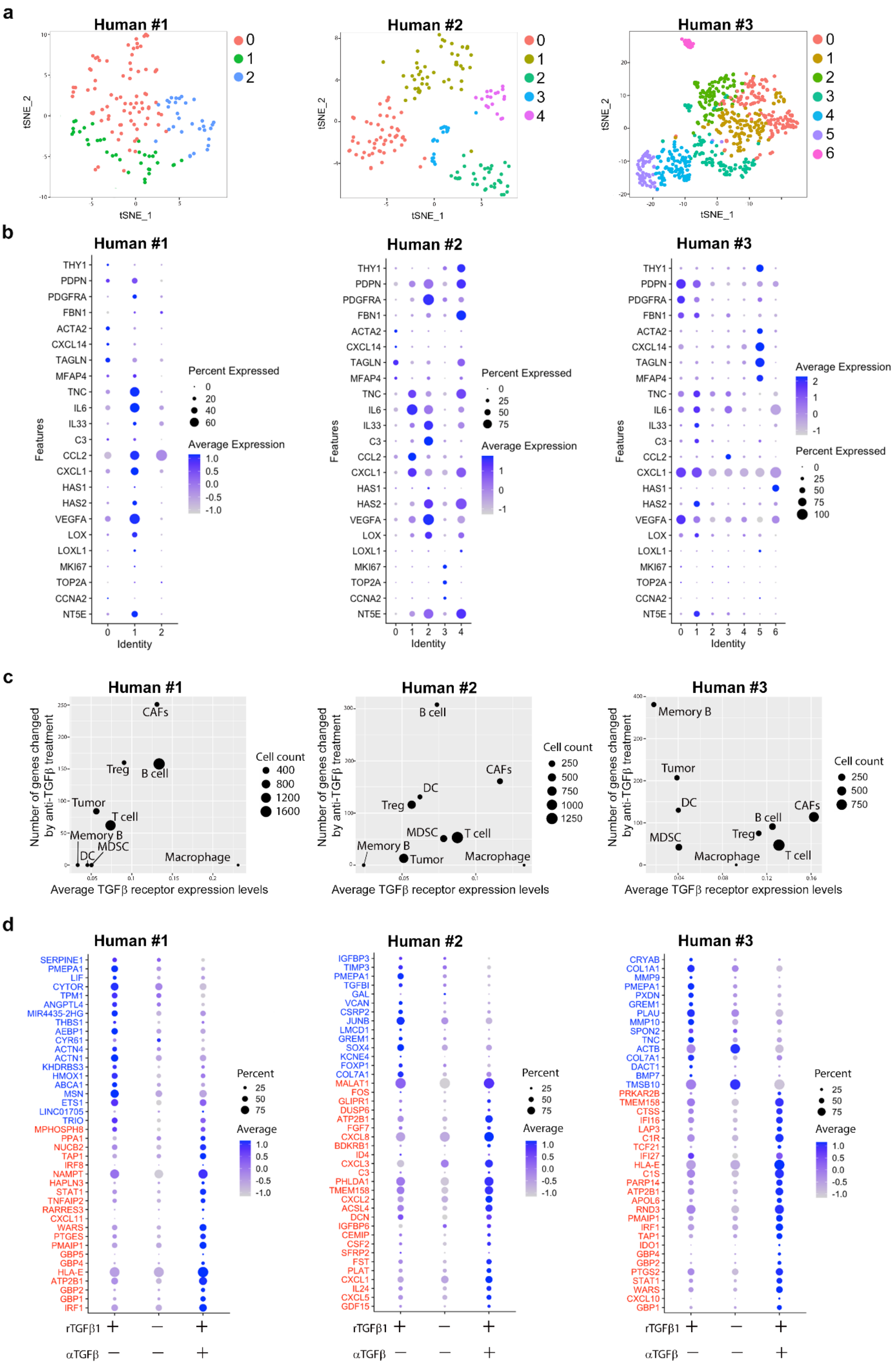

Supplementary Figure 7. Characterization of the response to TGFβ-neutralization in primary human CAFs. a. Baseline single cell RNAseq analysis of CAFs from cultures of dissociated primary human CRC tumors. Each plot represents independent clustering analysis from one individual. **b.** Bubbleplots depicting human sample expression of selected genes found to be differentially expressed among CAF subsets in 4T1 tumors. **c.** Graphical representation of the magnitude of transcriptomic alterations by cell type in human CRC tumors treated ex vivo with anti-TGFβ neutralizing antibody. **d.** Bubbleplots depicting the top 40 most differentially expressed genes following ex vivo TGFβ neutralization for each individual CRC tumor. Genes labeled in blue indicate downregulation by TGFβ neutralization, while genes labeled in red indicate upregulation.

Grauel et al., Supplementary Figure 8

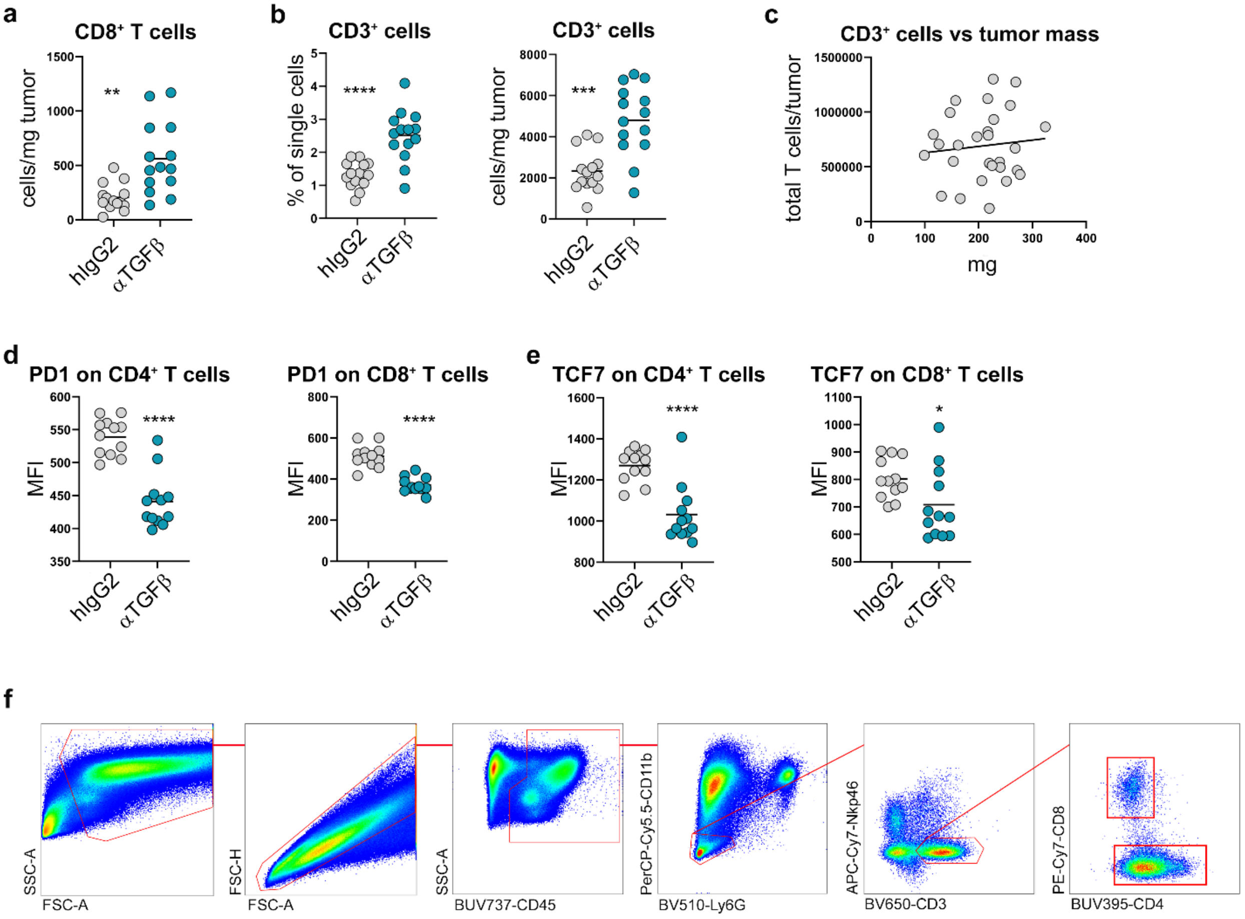

Supplementary Figure 8. Quantification and phenotype analysis of tumor infiltrating T cells following TGFβ neutralization. a. Flow cytometric analysis CD8⁺ T cells from 4T1 tumors treated with isotype- or anti-TGFβ normalized to tumor weight. Each dot represents a mouse. Mean is depicted. n=14 mice per group. Data are representative of three independent experiments. **p=0.0015 (unpaired, two-tailed t test with Welch's correction). **b.** Flow cytometric analysis of total T cells from 4T1 tumors treated with isotype or anti-TGFβ normalized to single cells and tumor weight. Each dot represents a mouse. Mean is depicted. n=14 mice per group. Data are representative of three independent experiments. ****p<0.0001; ***p=0.0002 (unpaired, two-tailed t test with Welch's correction). **c.** Total T cell counts per tumor plotted against total tumor mass. T cells per tumor calculated from flow cytometric analysis. **d, e.** Flow cytometric analysis of PD1 (**d**) and TCF7 (**e**) expression in CD4⁺ and CD8⁺ T cells from dissociated 4T1 tumors. Each dot represents a mouse. Mean is depicted. n=12 mice per group from one experiment. ****p<0.0001 and *p=0.0423 (unpaired, two-tailed t test with Welch's correction). **f.** Representative gating strategy for T cell flow cytometric analyses.

Grauel et al., Supplementary Figure 9

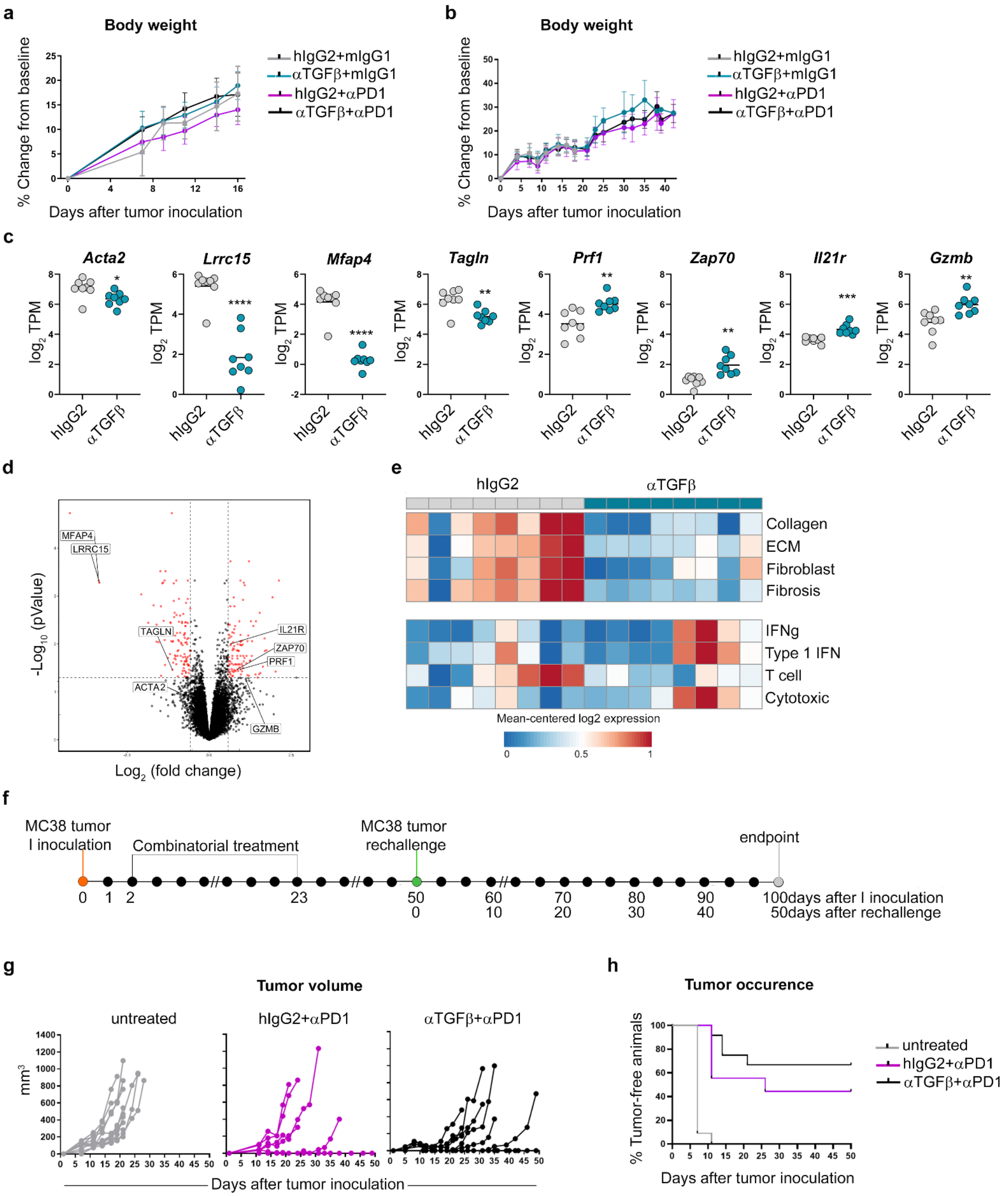

Supplementary Figure 9. TGFβ/PD1 co-blockade sustains development of immunological memory. a, b. Body weights from 4T1 (**a**) or MC38 (**b**) tumor-bearing mice treated with isotypes, anti-TGFβ, anti-PD1 or the combination of the two drugs were recorded throughout the study. Data are represented as mean +/- SD. n=15 mice per group. Data are representative of four independent experiments. **c-e.** Tumors from MC38-bearing mice treated with isotype control or TGFβ-blocking antibodies were pulverized and RNA was extracted for RNAseq analysis. **c.** Comparison of log2 TPM from bulk RNAseq data for selected genes in isotype and anti-TGFβ-treated mice. Each dot represents a mouse. n=8 mice per group. Data are representative of two independent experiments. p values for each marker are as follows: *Acta2* *p=0.0224; *Lrrc15* ****p<0.0001; *Mfap4* ****p<0.0001; *Tagln* **p=0.0028; *Prf1* **p=0.0030; *Zap70* **p=0.0017; *Il21r* ***p=0.0003; *Gzmb* **p=0.0050 (unpaired, two-tailed t test with Welch's correction). **d.** Volcano plot of bulk RNAseq data depicting changes in gene expression between treatments; horizontal dashed line indicates an adjusted p value of 0.05, vertical dashed lines indicate an absolute log2 fold change of 1 (p values were calculated based on a t-statistic for coefficients from a linear model fit to the data). **e.** Heatmap of stroma- and immune-associated signatures in bulk RNAseq data. Values were TPM normalized, log2 transformed, and row mean centered (z-score). Each column represents a mouse. **f.** Study schematic of MC38 re-challenge in vivo experiment. **g.** Tumor volume over time following re-challenge with MC38. Each line represents a mouse. **h.** The frequency of tumor-free mice over time after re-challenge. n= 8 (hIgG2) or 13 (αTGFβ) mice per group. Data are representative of two independent experiments.
